# Metal organic framework based synergistic improvement of hypoxia for optimizing diabetic wounds healing

**DOI:** 10.1016/j.mtbio.2026.103169

**Published:** 2026-04-30

**Authors:** Yunxian Dong, Lei Ren, Xiaoling Cao, Zhongye Xu, Zhongping Zhang, Jian Wang, Shiqi Wang, Zirui Zhao, Dongming Lv, Yongqing Li, Hui Fu, Zhigang Meng, Jia Tao, Peng Zhao, Bing Tang, Qing Tang

**Affiliations:** a Department of Plastic and Reconstructive Surgery, The First Affiliated Hospital of Sun Yat-sen University, Guangzhou, 510080, China; b Department of Breast Surgery, Shandong Cancer Hospital and Institute, Shandong First Medical University and Shandong Academy of Medical Sciences, Jinan, 250117, China; c Department of Burns, Wound Repair and Reconstruction, The First Affiliated Hospital of Sun Yat-sen University, Guangzhou, 510080, China; d School of Chemistry and Chemical Engineering, South China University of Technology, Guangzhou, 510640, China; e Guangdong Provincial Key Laboratory of New Drug Screening, School of Pharmaceutical Sciences, Southern Medical University, Guangzhou, 510515, China

**Keywords:** Diabetic wound, Metal organic framework, Wound healing, Macrophage polarization, Reactive oxygen species

## Abstract

In the physiological process of wound healing, an appropriate level of inflammation is conducive to the clearance of necrotic tissue and cells. However, due to insufficient blood supply and a high-glucose microenvironment, diabetic wounds have long been in a state of severe hypoxia and inflammation, which seriously hampers their transition to the proliferative stage. This study puts forward a strategy to synergistically ameliorate hypoxia based on ZnO_2_/CeO_2_@ZIF-8 (ZCZ). Specifically, ZnO_2_ generates exogenous O_2_ upon reacting with H_2_O, while CeO_2_ converts endogenous reactive oxygen species (ROS) into O_2_. During the early-stage repair processes, ZCZ can promote the proliferation, migration, and tubular formation of vascular endothelial cells, as well as neovascularization. Through an in vivo diabetic wound model, it was observed that ZCZ significantly reduced the wound healing time. It also enhanced the dermal thickness by increasing collagen deposition, along with elevating the presence of M2 macrophages, augmenting neovascularization, and improving the oxygen-carrying capacity. Mechanism studies have indicated that ZCZ mainly exerts its effects through inhibiting the IL6/JAK2/STAT3 pathway. Consequently, the proposed strategy not only offers a novel clinical option but also paves a new path for the management of chronic and recalcitrant wounds.

## Graphical abstract

**Figure:**
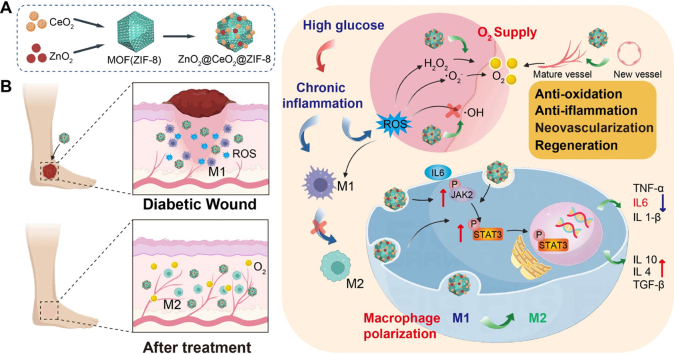

## 1 Introduction

Diabetic wound is one of the most prevalent and severe chronic complications among diabetic patients, characterized as a typical chronic refractory wound [1,2]. Despite standardized treatment, only 30% of diabetic wounds heal after 5 months. Therefore, developing an effective treatment strategy to promote diabetic wound healing is crucial [3]. The physiological healing process of wounds consists of four stages: hemostasis and coagulation, inflammation, proliferation, and remodeling [4]. During the initial stages of hemostasis and early inflammation, macrophages polarize into classically activated (or inflammatory) macrophages (M1), enhancing the clearance of necrotic tissue and cells on the wound surface by secreting inflammatory cytokines and chemokines [5,6]. In the late inflammatory and proliferative stages, macrophage polarize into alternatively activated (or wound-healing) macrophages (M2), which inhibit the inflammatory response and promote tissue repair by secreting anti-inflammatory factors and growth factors [[7], [8], [9]]. However, diabetic wounds are characterized by high glucose levels, hypoxia, and excessive reactive oxygen species (ROS), leading to the obstruction of macrophage polarization to the M2 phenotype and the persistence of M1 [10,11]. The wound remains in the inflammatory stage for an extended period and fails to transition to the proliferative stage [12]. Therefore, reducing the inflammatory response caused by oxidative stress, promoting angiogenesis, and improving tissue oxygen content are key to promoting diabetic wound healing.

In recent years, metal oxides and metal peroxides have displayed great potentials in regulating the microenvironment of disease [13]. Zinc ions can induce angiogenesis, and exhibit anti-inflammatory and antioxidant abilities, making them a robust tool for treating ischemic diseases of the lower limbs [14,15]. ZnO nanoparticles have traditionally been used to promote wound healing [15,16]. Compared to ZnO, Zinc peroxide (ZnO_2_) has the advantage of carrying exogenous oxygen (O_2_) as it can react with water to generate O_2_ [17,18]. Cerium dioxide (CeO_2_) has the characteristic of mimicking multiple antioxidant enzymes, such as superoxide dismutase (SOD) and catalase, which can convert O_2_^•−^ and H_2_O_2_ into oxygen (O_2_), respectively [[19], [20], [21]]. Based on this analysis, it is speculated that the combination of ZnO_2_ and CeO_2_ can synergistically improve the microenvironment of diabetic wounds by co-delivering exogenous and endogenous oxygen, reducing oxidative stress, promoting angiogenesis.

Apart from the active subject, a proper carrier is necessary to design a nano-materials with high stability and efficiency. Metal-organic frameworks (MOFs) are a class of 2D or 3D porous organic-inorganic hybrid crystalline materials where the metal center and organic ligand are interconnected through self-assembly, such as zinc imidazole framework-8 (ZIF-8), etc [22]. In recent decades, MOFs have garnered significant attention in biomedical research, including biosensing, drug delivery, magnetic resonance imaging, and disease treatments [23,24]. The outstanding performances of MOFs are attributed to their structure-related multiple properties [25,26]. Firstly, they have high drug loading capacity due to their large surface area and high porosity [27]. Secondly, the metal ions (or metal clusters) and ligands are easily designed and modified, endowing MOFs with good biocompatibility and controlled release of active substances [23,28]. Thirdly, MOFs can participate in the composition of drug transport systems, allowing them to be triggered under various specific conditions (heat, magnetic, pH, and redox, etc.) [29,30]. Thus, MOFs can serve as powerful tools for co-delivering ZnO_2_ and CeO_2_ nanoparticles.

**Scheme 1:**
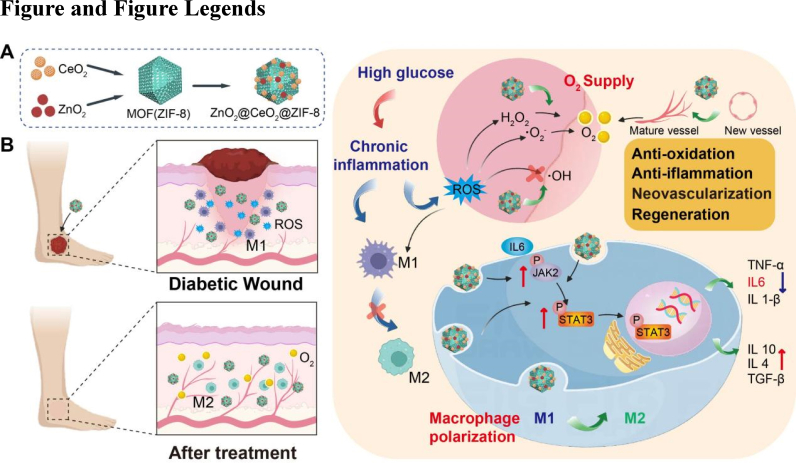
**Schematic illustration of the preparation of ZCZ, and the effect on diabetic wound treatment.** (A) Illustration of the ZCZ synthesis method. (B) Schematic illustration of ZCZ for diabetic wound healing by M2 macrophage polarization, increasing oxygen supply, and promoting vascularizaion.

Herein, a novel nanocomposite ZnO_2_/CeO_2_@ZIF-8 (ZCZ) was constructed via hydrothermal reactions intended to improve diabetic wound healing (Scheme 1). In vitro experiments revealed that ZCZ is effective in providing endogenous and exogenous oxygen, eliminating ROS, improving the proliferation and migration of human umbilical vein endothelial cells (HUVECs), and inducing phenotypic polarization of macrophages. Specifically, in a high-glucose environment, ZCZ could enhance the survival ability of HUVECs, inhibit the expression of proinflammatory cytokines (IL1β, TNF-α, IL6), and promote the expression of pro-repair cytokines (IL4, IL10, TGF-β) in RAW 246.7 cells. ZCZ with a higher ratio of CeO_2_/ZnO_2_ (Z_1_C_2_Z) performed better than that with a lower ratio of CeO_2_/ZnO_2_ (Z_1_C_1_Z). In vivo, after injecting the nano-materials around the wounds of db/db mice, the Z_1_C_2_Z group achieved a wound healing rate of 98% on day 21, compared to 52.7% in the control group. Pathology and photoacoustic imaging results suggested that the ZCZ group achieved better healing quality, including lower inflammatory factors, increased M2 macrophage polarization, enhanced neovascularization, and higher oxygen supply. Mechanistically, RNA sequencing further revealed that ZCZ promotes wound healing through the IL6/JAK2/STAT3 pathway.

## 2 Methods and materials

### 2.1 Materials and reagents

Polyvinyl Pyrrolidone (PVP), Ce(NO_3_)_3_, ethanol, Zn(NO_3_)_2_, 2-methylimidazole (2-MI), methanol, polyethylene glycol (PEG), NaNO2, HCl, NaOH, KO_2_, NH_4_OH, H_2_O_2_, 5-tert-Butoxycarbonyl-5-methyl-1-pyrroline N-oxide (BMPO), 18-crown-6, Rhodamine B, diethyl ether and dimethyl sulfoxide (DMSO) were purchased from Macklin (Shanghai, China). 1 × DMEM medium, fetal bovine serum (FBS) and tris(4,7-diphenyl-1,10-phenanthroline) ruthenium II dichloride complex (Ru(dpp)_3_Cl_2_ were purchased from Gibco (New York, USA). Endothelial cell medium was purchased from ECM, ScienCell (USA). 4% paraformaldehyde, Triton- × 100, goat serum and rat tail collagen were purchased from Solarbi (Beijing, China). Cell Counting Kit-8 (CCK8) kit, phenylmethylsulfonyl fluoride (PMSF), radioimmunoprecipitation assay (RIPA) buffer and Bicinchoninic Acid (BCA) protein assay kit was purchased from Fudebio (Hangzhou, China). Collagenase type II was purchased Yuanye (Shanghai, China). Cell live-dead kit and 4,6-diamidino-2-phenyiindole 2 hci (DAPI) were purchased from keyGEN (Nanjing, China). Annexin V-FITC/PI apoptosis kit, TUNEL in situ apoptosis Kit (Green, FITC), reactive oxygen species (ROS) fluorometric assay kit (2,7-dichlorofluorescein, DCFH-DA), mitochondrial membrane potential assay kit (with JC-1) and mitochondrial extraction assay kit were purchased from Elabscience (Wuhan, China). Polyvinylidene difluoride (PVDF) membranes was purchased from Roche (Mannheim, Germany). Citric acid antigen recovery buffer, 2.5% glutaraldehyde, hydrogen peroxide solution, hematoxylin-eosin (HE) high definition constant dye kit, Masson Tri-Color dyeing solution, Picrosirius Red dye and immunohistochemical kit were purchased from Servicebio (Wuhan, China). For immunofluorescence staining, the primary antibodies included γ-H2AX (CST, USA, #9718), HIF-1α (CST, USA, #36169), iNOS (CST, USA, #13120 and Abcam USA, ab209027), CD206 (CST, USA, #24595), F4/80 (Bioss, China, bsm-34028M). Enzyme linked immunosorbent assay (ELISA) kits of TNF-α, IL-1β, IL-6, IL-4, TGF-β1, IL-10 were purchased from BYabscience (Nanjing, China). Hydroxyproline colorimetric assay kit was purchased from BioVision (San Francisco, USA).

### 2.2 Preparation of ZnO_2_@CeO_2_@ZIF-8

Firstly, 500 mg of Zn(NO_3_)_2_ and 1g PVP were mixed under vigorous strring in 60 mL ethanol. Next, 5 mL of NH_4_OH (0.8 M) was added and 1 mL H_2_O_2_ (1M) was added drop by drop. After 1.5 h, the ZnO_2_ nanoparticles were separated by centrifugation and washed with ethanol.

CeO_2_ nanoparticles were synthesized using wet chemistrymethods. Briefly, the Ce(NO_3_)_3_ was dissolved in deionized water, the solution was oxidized using excess hydrogen peroxide. After 1.0 h, the CeO_2_ nanoparticles were separated by centrifugation and washed with ethanol.

5 mg ZnO_2_ nanoparticles and 5 mg (or 10 mg) CeO_2_ nanoparticles were added to 10 mL of methanol and sonicated for 10 min. Then, 0.5 g 2-methylimidazole and 0.1 g Zn(NO_3_)_2_ were added to the solution and reacted at room temperature for 30 min. The prepared ZnO_2_@CeO_2_@ZIF-8 (ZCZ) was separated by centrifugation, washed with methanol and finally dispersed in 10 mL methanol. For the ZCZ with different ratio of ratio of CeO_2_/ZnO_2_, 5 mg and 10 mg CeO_2_ nanoparticles were used to synthesize Z_1_C_1_Z and Z_1_C_2_Z, respectively.

### 2.3 Characterization of ZCZ

The surface morphology, elemental distribution of ZCZ were analyzed using high-resolution transmission electron microscopy (HRTEM, JEM-2100F, JEOL, Japan). The X-ray photoelectron spectroscopy (XPS) was measured by X-ray photoelectron spectrometer (ESCALAB-250Xi, Thermo, USA). The Fourier transform infrared (FT-IR) spectra was obtained using an infrared spectrometer (Nicolet-6700, Thermo, USA). Dynamic light scattering and ζ potential of the nanoparticles were measured using Malvern Zeta Sizer Nano series (Nano ZS, Malvern Instruments, U.K.).Ultraviolet-visible-near-infrared spectra of NPs were measured by spectrophotometer (PerkinElmer Lambda 750, USA).

### 2.4 ROS-scavenging ability

CAT-like ability studies were performed with the UV-vis absorption spectra (UV-5500PC, METASH, China) to obtain absorbance of H_2_O_2_ solution in the absence and presence of CeO_2_ and ZCZ. In a typical assay, H_2_O_2_ solution (200 μL, 40 mM) mixed with different materials (20 μL) was added into the colorimetric dish and then performed UV-vis absorption spectra. Absorbance of H_2_O_2_ solutions at different concentration without and with nanozymes was monitored at 351 nm.

SOD-like ability studies were implemented as manufacturer's instructions describe in the SOD assay kit (Elabscience, Wuhan, China). Absorbance at 450 nm was detected by UV-Vis spectrum for further SOD activity ascertaining.

### 2.5 RNS-scavenging ability

For ONOO^−^ generation, NaNO_2_ solution (10 mL, 50 mM) was mixed with H_2_O_2_ (10 mL, 25 mM) under stirring in a 50-mL beaker for 3 min. Then HCl (5 mL, 1 M) and NaOH (5 mL, 1.5 M) were poured in sequence within 1 s. And stirring was keeping on until the reaction mixture turns from transparent into light yellow. The entire process was operated under an ice-water bath. The resulting solution was diluted suitably to obtain the characteristic peak of ONOO^−^ at 302 nm. Then, CeO_2_ or ZCZ (3 mg/mL, 10 μL) were added to the weak solution (190 μL) to observe the absorption at 302 nm with the reaction time.

For DPPH• radical scavenging activity, different concentrations of ZCZ solutions (0∼800 μg/mL) and HAc/NaAc buffer (180 μL. 100 mM) were added to DPPH ethanol solution (0.3 mM, 200 μL) and whirls violently in the dark. The UV absorbance was monitored at 517 nm every 5 min within 40 min, and the detection range of UV spectra was 400-700 nm.

### 2.6 Cell culture

The human umbilical vein endothelial cells (HUVECs) and Raw 264.7 cells were acquired from Shanghai Zhong Qiao Xin Zhou Biotechnology Co.,Ltd. Raw 246.7 cells were cultured in DMEM supplemented with 10% FBS at 37 °C in 5% CO_2_. HUVECs were cultured in ECM supplemented with 10% FBS (Gbico) at 37 °C in 5% CO_2_. For experimental treatment, cells were subjected to normal glucose (NG, 5 mmol/L) or high glucose (HG, 30 mmol/L) for further experiments.

### 2.7 Cellular uptake ability assay

HUVECs were seeded in a confocal glass bottom dish at a density of 2000 cells and cultured for 24 h. After the HUVECs incubated with Rhodamine B-conjugated Z_1_C_1_Z and Z_1_C_2_Z for 1.5, 3 and 6 h, the cells were fixed with 4% paraformaldehyde, sequentially stained with DAPI, and finally observed with a confocal laser scanning microscopy (CLSM, LSM880 Basic Operation, Zeiss).

### 2.8 In vitro cytotoxicity and cell viability assay

HUVECs and Raw 264.7 cells (2 × 10^3^ cells/well) were cultured in 96-well plates overnight and treated in triplicate with different doses of Z_1_C_1_Zand Z_1_C_2_Z (0, 10, 25, 50, 100, 200 μg/mL) for 12-48 h. The nano-materials were dissolved in phosphate buffer solution (PBS). The cell viability was determined using a CCK8 kit per the manufacturer's protocol. We stained the cells by Calcein/PI for 10 min and observed the morphology of the cells by confocal microscopy (LSM880 Basic Operation, Zeiss, Germany).

### 2.9 Intracellular ROS production detection

The impact of different nano-materials on ROS production in HUVECs was determined by flow cytometry and immunofluorescence. HUVECs were treated in triplicate with different nano-materials (Z_1_C_1_Z and Z_1_C_2_Z) at 37 °C for 48 h, then culture media was replaced with 500 μM of H2O2 for 2 h. Then the cells were cultured in serum-free DMEM in the presence of 10 μM DCFH-DA at 37 °C for 30 min in the dark. The cells were harvested for flow cytometry analysis in a Beckman Cyan flow cytometer (Beckman Coulter, Brea, CA, USA) and immunofluorescence staining.

### 2.10 Intracellular mitochondrial membrane potential detection

The impact of lycorine on mitochondrial potential of HUVECs was analyzed using JC1 staining. Briefly, HUVECs were treated in triplicate with 50 μg/mL of Z_1_C_1_Z and Z_1_C_2_Zat 37 °C for 48 h, then incubated with 500 μM of H2O2 for 2 h, and stained with JC-1 for 15 min at 37 °C in the dark. After being washed, the cells were analyzed under a fluorescence microscope (LSM880 Basic Operation, Zeiss).

### 2.11 Immunofluorescence staining

HUVECs and Raw 264.7 cells were cultured on glass coverslips in dishes overnight, and treated with different conditions for 48h. The cells were fixed with 4% paraformaldehyde for 30min, permeabilized with 0.25% of Triton-X100 for 20 min, blocked with goat serum for 1 h at room temperature. The cells were incubated with primary antibodies overnight at 4 °C. After being washed, the bound antibodies were reacted with fluorescent secondary antibodies at room temperature for 1 h and co-stained with DAPI. The fluorescent signals were captured under a confocal microscopy (LSM880 Basic Operation, Zeiss). Similarly, mice wound scar tissue sections were fixed in 4% paraformaldehyde and subjected to immunofluorescence. The concentration of all primary antibodys was 1:400.

### 2.12 Enzyme linked immunosorbent assay (ELSA)

Raw 264.7 cells were treated with M1 macrophage inducer (60 ng/mL LPS + 20 ng/mL INFγ) or M2-type macrophage inducer (20 ng/mL IL4) and different nano-materials (Z_1_C_1_Z and Z_1_C_2_Z) for 48 h in high glucose. Briefly, the cells were homogenized with RIPA buffer and centrifuged at 10,000 rpm for 15 min at 4 °C. The supernatant was collected and used for determination of the protein levels of TNF-α, IL-1β, IL-6, IL-4, TGF-β1, IL-10, using ELISA kits following the manufacturer's protocols.

In vivo, mouse wound skin was homogenized with RIPA buffer. The remaining procedures were the same as in vitro experiments. Determination of the protein levels of TNF-α, IL-1β, IL-6 at day 7 and IL-4, TGF-β1, IL-10 at day 14, respectively.

### 2.13 Wound healing assay

HUVECs were cultured in 6-well plates and when they reached 100% confluence, the monolayer of cells was wounded by a scratch using a pipette tip. After washing out the non-adherent cells, the remaining cells were cultured for 24 h in different conditions. The wound healing of cell monolayer in each well was photoimaged and the percent of wound healing was calculated using ImageJ software.

### 2.14 Transwell assay

The HUVECs were digested, centrifuged and resuspended in serum-free medium. 600 μL of medium containing 20% FBS was added to the lower chamber of the 24-well plate. There were five groups: low glucose, high glucose, high glucose with Z_1_C_1_Z and Z_1_C_2_Z respectively. Then, 200 μL of cell suspension was added to the transwell chamber (without gel). The high nutritional content of the medium in the lower chamber should promote cell migration. The number of cells entering the lower chamber reflects the invasion ability of cells. 2 × 10^3^ cells were seeded in each chamber. Cells were cultured for 24 h in different conditions. The next day, transwell chamber were taken out and non-migrated cells were wiped off. Then, chambers with cells were fixed with paraformaldehyde for 30 min and stained with 0.1% crystal violet for another 30 min. The number of cells in five visual fields was counted immediately under 10 × magnification.

### 2.15 Tube formation assay of HUVECs

The Matrigel was prelaid on the bottom of the 24-well plate at 4 °C, then the high-glucose medium was mixed with the three nano-materials to achieve a drug concentration of 50 μg/mL. Finally, HUVECs were resuspended seeded with 5 × 10^4^ cells per well into 24-well plate for 24 h. The tube in each well was photoed and quantified using ImageJ software.

### 2.16 Oxygen generation assay

HUVECs were treated in triplicate with different nano-materials (Z_1_C_1_Z and Z_1_C_2_Z) at 37 °C for 48 h, then culture media was replaced with 500 μM of H_2_O_2_ for 2 h. Then the cells were cultured in serum-free DMEM in the presence of 10 μM Ru(dpp)_3_Cl_2_ at 37 °C for 30 min in the dark. The cells were harvested for immunofluorescence staining.

### 2.17 Hemolysis test

A hemolysis test was performed to evaluate the blood compatibility of the samples [31]. Fresh blood was obtained from 10-weeks old db/db mice. Briefly, Z_1_C_1_Z and Z_1_C_2_Z were added to tubes containing physiological saline (0.5 mL). Diluted blood (0.5 mL) was added and the mixture was incubated at 37 °C. After 60 min, the solution was centrifuged at 1000 rpm for 15 min and the absorbance at 540 nm was recorded. DI water and PBS were selected as positive and negative groups, respectively. Hemolysis was measured using Equation (1):

(1) Hemolysis ratio (%) = [(OD_h_ - OD_n_) / (OD_p_ - OD_n_)] × 100%

where, OD_h_, OD_n_, and OD_p_ indicate the absorbance values of the sample, PBS, and DI water groups, respectively.

### 2.18 Animal wound model

**2.18.1 For C57BL mice** 6-weeks old C57BL mice were from Guangdong medical laboratory animal center. This study was approved by the Ethics Committee for Clinical Research and Animal Trials of the First Affiliated Hospital of Sun Yat-sen University (Guangzhou, China, approval number: lunshen [2021] 168). Thirty-six C57BL mice were anesthetized by inhaling isoflurane, and two circular 10-mm full-thickness wounds were created on their dorsum. The wounds were divided into three groups randomly: control group (PBS), Z_1_C_1_Z group and Z_1_C_2_Z group. Following the wounded, the wound healing process were monitored longitudinally. Three rats of each group were sacrificed at days 0, 7, 14, and 21, respectively, and the wound tissues were harvested for histological analysis.

2.18.2**For diabetic db/db mice** 10-weeks old db/db mice were from Guangdong medical laboratory animal center. Thirty-six db/db mice were anesthetized by inhaling isoflurane, and two circular 10-mm full-thickness wounds were created on their dorsum. The wounds were divided into three groups randomly: control group (PBS), Z_1_C_1_Z group and Z_1_C_2_Z group.

Following the wounded, the wound healing process were monitored longitudinally. Three rats of each group were sacrificed at days 0, 7, 14, and 21, respectively, and the wound tissues were harvested and separated into two halves across the center: one half was processed for histological, immunohistochemistry and immunofluorescent analysis, and the other was rapidly frozen in liquid nitrogen for ELISA and protein analysis.

### 2.19 Transcriptomics and bioinformatics analysis

The effect of ZCZ on gene transcription in mouse skin wounds tissue was determined by RNA-seq and bioinformatics techniques performed at oebiotech (Shanghai, China). Briefly, skin tissue from mice wounds was taken from mice treated with ZCZ for 15 days. And their total RNA was extracted using the RNA Easy Fast Tissue/Cell kit (TIANGEN, China), followed by RNA sequencing. The differentially expressed genes (DEGs) were determined by a fold-change of 2 or p < 0.05. The distribution of DEGs was presented by the volcano map and the top DEGs were exhibited by a heatmap. The potential functions of DEGs were analyzed by Gene Ontology (GO) enrichment analysis and Kyoto Encyclopedia of Genes and Genomes (KEGG) using the clusterProfiler R package (3.4.4) as well as by GSEA (http://www.broadinstitute.org/gsea/). Raw data are deposited in the Gene Expression Omnibus database under accession code GSE324514 (https://www. ncbi.nlm.nih.gov/geo/).

### 2.20 Histopathology

The dissected wound tissues were fixed in 10% of formalin overnight and paraffin-embedded. The tissue sections (5 μm) were regularly stained with hematoxylin and eosin and masson staining. The stained tissue sections were captured under a light microscope (Digital pathology section scanner, KFBIO, China, KF-PRO-020-HI).

### 2.21 Hydroxyproline assay

The hydroxyproline content of the wound skin tissue was utilized to assess collagen, and measured by the Hydroxyproline Colorimetric Assay Kit (BioVision, San Francisco, CA, USA) according to the manufacturer's instructions. Briefly, wound skin tissue was hydrolyzed, oxidized by H_2_O_2_ and colored by p-dimethylaminobenzaldehyde, and absorbance was tested at 540 nm. The amount of Hydroxyproline was expressed as mg/g tissue.

### 2.22 Photoacoustic imaging

For functionalized blood vessel evaluation, mouse wounds were scanned on day 21 using the Multispectral Optoacoustic Tomography inVision system (iThera Medical, Germany). The percentages of average hemoglobins and oxygenated hemoglobin percentages in total in the regenerative tissues were quantified using the ImageJ tools.

### 2.23 Statistical analysis

The data are expressed as mean ± SD. The difference between two groups was analyzed by the unpaired *t*-test analysis and the difference among multiple groups was analyzed by one-way ANOVA and post hoc Bonferroni's correction using SPSS 24.0 software. P-value of <0.05 was considered statistically significant.

## 3 Results

### 3.1 The characterization of ZCZ

**Fig. 1:**
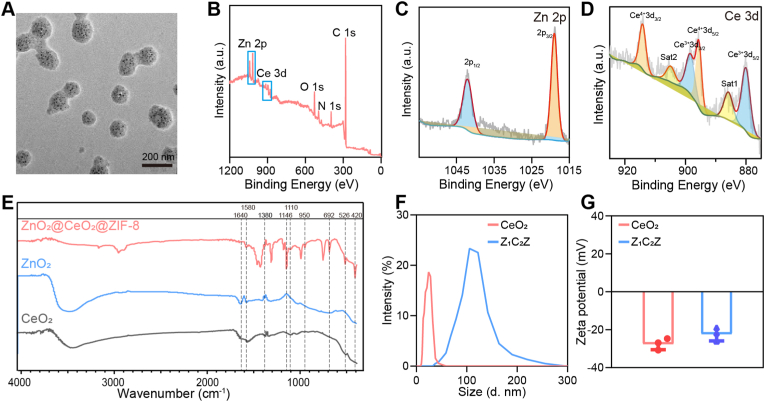
Characterization of ZCZ. (A) TEM of ZCZ, Scale bar: 200 nm. (B) XPS of ZCZ. (C) Zn 2p and D) Ce 3d spectra of ZCZ. The (E) FTIR spectra, (F) hydrodynamic diameter distribution and (G)zeta potential of different nanoparticles.

Transmission electron microscopy (TEM) results indicated that the ZCZ had a size of about 100 nm, with CeO_2_ and ZnO_2_ nanoparticles uniformly distributed in ZIF-8 (Fig. 1A). The X-ray photoelectron spectroscopy (XPS) spectrum for core level peaks of C 1s, N 1s, O 1s, Ce 3d, and Zn 2p electrons in ZCZ are illustrated in Fig. 1B. The XPS spectrum for Zn 2p showed two peaks at 1042.1 eV (1/2) and 1019.0 eV (1/2) (Fig. 1C). XPS analysis also confirmed the four characteristic peaks of Ce^3+^ and Ce^4+^ (Fig. 1D). The peaks centered on 914.2 eV and 895.6 eV belong to 3d3/2 and 3d_5/2_ states of Ce^4+^. The 3d_3/2_ and 3d_5/2_ states of Ce^3+^ are located at 898.6 eV and 880.2 eV. In addition, two satellite peaks appear at 892.3 eV and 907.6 eV, respectively [32]. The peaks centered on 914.2 eV and 895.6 eV belong to 3d_3/2_ and 3d_5/2_ states of Ce^4+^. The 3d_3/2_ and 3d_5/2_ states of Ce^3+^ are located at 898.6 eV and 880.2 eV. In addition, two satellite peaks appear at 892.3 eV and 907.6 eV, respectively [32,33]. Fourier transform infrared (FTIR) spectra showed two characteristic peaks at 420 cm^−1^ and 526 cm^−1^, which belong to ZnO_2_ (Zn-O) and CeO_2_ (Ce-O) respectively (Fig. 1E) [34,35]. And the vibration peak at 1380 cm^−1^ for the peroxy bond. The ZIF-8 exhibits characteristic peaks (Fig. 1E) at 1580 cm^−1^ (corresponding to C=N elongation vibration), in the range of 1300 to 1460 cm^−1^ (indicating aromatic ring vibrations), at 1146 cm^−1^ and 950 cm^−1^ (representing stretching of the aromatic group C-N bonds). Furthermore, the characteristic peaks at 735-760 cm^−1^ are associated with the folding of the aromatic group C-H bond, while the peaks at 692 cm^−1^ are attributed to the imidazole ring of the organic ligand (Fig. 1E) [36,37].The hydrodynamic diameters of CeO_2_ and ZCZ were determined to be 22.63 and 110.04 nm, respectively (Fig. 1F). The zeta potential of CeO2 and ZCZ was detected as −27.5 mV and −22.3 mV (Fig. 1G).

### 3.2 The antioxidant enzyme simulated ability of ZCZ

The ZnO_2_ was used due to two reasons, the release of Zn^2+^ and H_2_O_2_, the latter can serve as an oxygen carrier with the existence of antioxidant enzyme, such as catalase (CAT) [38]. Therefore, the H_2_O_2_ production capacity of ZnO_2_ was measured by potassium permanganate method [39]. As shown in Fig. S1, it was observed a proportional increase of H_2_O_2_, which was resulted from the reaction of ZnO_2_ with H_2_O. Then, the CAT-like activity of CeO_2_ and ZCZ was determined by UV-visible spectroscopy. With the increase of the amount of CeO_2_ (0-1000 μg/mL) added to the system, the more H_2_O_2_ was decomposed, the lower the absorption peak value at 351 nm (Fig. S2A and S2B). In Fig. S2C and S2D, the peak intensity at 351 nm also decreased with the increase of ZCZ concentration, which was mainly due to the CAT-like activity of CeO_2_ on its surface.

The SOD-like activity of ZCZ was subsequently examined (Fig. S3), it can be seen that the absorption intensity at 450 nm decreased with the ZCZ concentration increased from 112.5 μg/mL to 890 μg/mL, confirming its SOD-like activity [40]. Finally, the capacity of ZCZ for the removal of reactive nitrogen species (RNS) including ONOO^−^ was investigated. As evidenced in Fig. S4, the absorption at 302 nm gradually decreased with increasing ZCZ concentration of 0-800 μg/mL. The scavenging ability of ZCZ against DPPH, a stable organic free radical, has also been confirmed, as illustrated in Fig. S5.

### 3.3 Biocompatibility of ZCZ

**Fig. 2:**
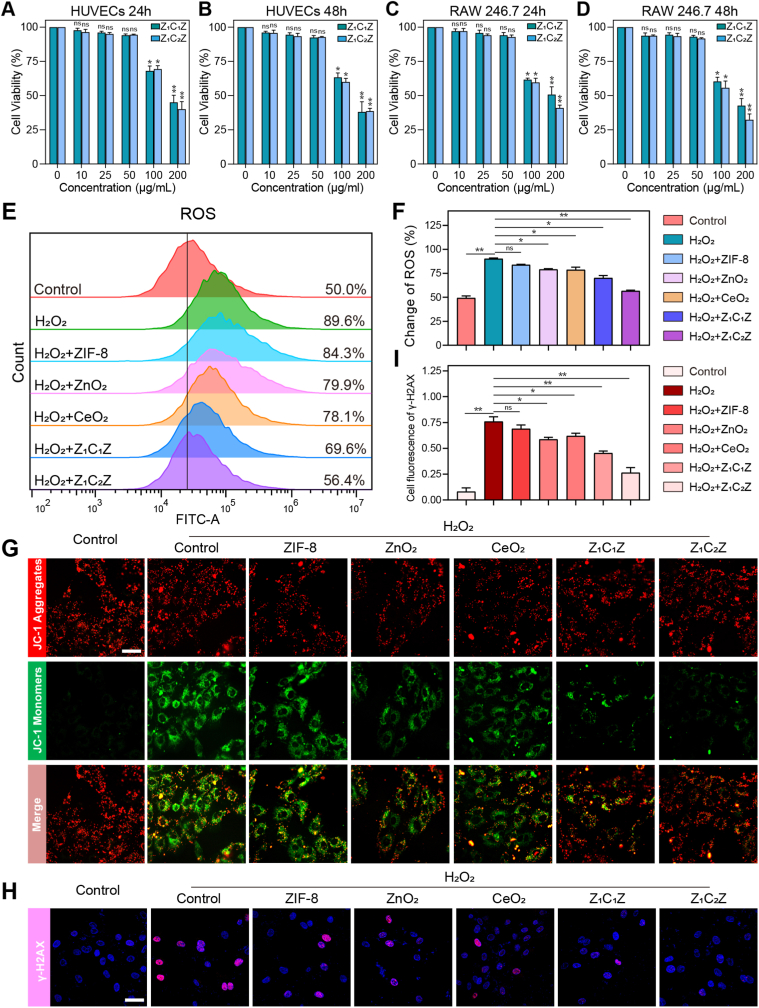
Nano-materials attenuated H_2_O_2_-induced ROS generation and oxidative stress damage in HUVECs. (A-D) Cell survival of HUVECs and RAW246.7 treated withZ_1_C_1_Zand Z_1_C_2_Z at concentrations from 0 to 200 μg/mL at 24 h and 48 h, n = 3. (E, F) Flow cytometry analysis of ROS with DCFH-DA probe in HUVECs with different treatments for 24 h, n = 3. (G) Representative images of mitochondrial membrane potential detected by JC-1 fluorescent probe, n = 3. Scale bar: 20 μm. (H, I) Immunofluorescence photos for the effect of different nano-materials on the expression of γ-H2AX, n = 3. Scale bar: 20 μm. Data are mean standard error of the mean, p values are based Student's *t*-test. ∗p < 0.05, ∗∗p < 0.01, ∗∗∗p < 0.01, ns, nonsignificant.

Before the further medical application, the cytocompatibility of ZCZ nanoparticles was evaluated. Firstly, the Human umbilical vein endothelial cells (HUVECs) were treated with Rhodamine B-labeled ZCZ, and fluorescent images in Fig. S6 demonstrated the cellular uptake of ZCZ at 0, 3, and 6 h. Then, HUVECs and macrophage RAW 264.7 were treated with a gradient concentration of 0-200 μg/mL at 24 h and 48 h respectively by CCK8 assay. As shown in Fig. 2A and B, after 24 h of treatment, the survival rates of HUVECs at 50 μg/mL were 94.0% (Z_1_C_1_Z) and 92.7% (Z_1_C_2_Z), and the cell viability of RAW264.7 was also >90% (Fig. 2C and D). When the concentration ofZ_1_C_1_Zand Z_1_C_2_Z was increased to 100 μg/mL, the cell vitality of HUVECs decreased to 68.0% and 69.3%, and that of RAW264.7 was 61.7% and 59.7%, respectively. Therefore, 50 μg/mL was selected as the optimized concentration for subsequent experiments. Further, HUVECs were stained with calcein AM (2 μM) and propidium iodide PI (8 μM), red fluorescence was almost invisible at 50 μg/mL, also indicating the well cytocompatibility (Fig. S7). Before the in vivo experiments, the biosafety of ZCZ was evaluated through hemolysis assay. Fig. S8 showed that neitherZ_1_C_1_Znor Z_1_C_2_Z can induce hemolysis of red blood cells of C57 mouse, indicating its well biosafety.

In vivo, H&E staining results of major organs of both C57 and db/db diabetic mice in different groups showed no significant difference between the control group and the treatment groups, and no damage, infection or inflammation of heart, liver, spleen, lung and kidney were observed (Figs. S9 and S10). In addition, there was no significant difference in body weight between the experimental and control groups for C57 and db/db mice (Figs. S11 and S12).

### 3.4 ZCZ protected cells from oxidative damage

In diabetic wounds, the high level of blood glucose leads to the oxidative stress microenvironment, which is not conducive to HUVECs proliferation and seriously affects the wound healing efficiency [41,42]. Based on the efficient free redical removal effect of ZCZ, we then examined the ability of ZCZ for protecting HUVECs from oxidative damage. HUVECs were treated with H_2_O_2_ (5 mM) to simulate the oxidative stress damaged microenvironment. The severity of oxidative stress damage can typically be ascertained by directly detecting the level of ROS in cells. As shown in Fig. 2E and F, the ROS level in the H_2_O_2_ treatment group was significantly higher than that in the blank control group. Moreover, mitochondria is the main effector organelles of ROS, recent studies reported that the removal of mitochondrial ROS by antioxidants significantly accelerated skin wound healing in diabetic mice. Therefore, JC-1 probe was used to label mitochondria, in which the ratio of green and red fluorescence could indicate the change of mitochondrial membrane potential (MMP). In the MMP change detection (Fig. 2G), the green-red fluorescence ratio in the control group was 0.06, indicating that the mitochondria had a normal MMP, while in the H_2_O_2_ group, the green fluorescence became stronger and the ratio increased to 1.32. The above data have confirmed that HUVECs treated with H_2_O_2_ (5 mM) are unequivocally in a state of oxidative stress damage.

Compared to the control group, H_2_O_2_ caused 30% of HUVECs to die, and the proportion of dead cells decreased after pretreatment with 50 μg/mL concentrations of Z_1_C_1_Z and Z_1_C_2_Z (Fig. S13A and B). Next, we investigated ROS levels in HUVECs treated with different materials co-incubated with H_2_O_2_. DCFH-DA (2′, 7′- Dichlorodihydrofluorescein diacetate) was used to detect the levels of ROS. After H_2_O_2_ (5 mM) treatment, ROS generation in HUVECs increased significantly. Initially, we investigated the effects of ZIF-8, ZnO_2_, and CeO_2_ on ROS scavenging. ZIF-8 alone had a negligible effect on ROS scavenging, while ZnO_2_ and CeO_2_ alone could slightly reduce the level of ROS (Fig. 2E and F). Interestingly, pretreatment of Z_1_C_1_Z and Z_1_C_2_Z significantly promoted ROS clearance respectively (Fig. S14). Further flow cytometry showed that the ROS scavenging capacity of Z_1_C_1_Z and Z_1_C_2_Z was 19.8% and 33.2%, respectively (Fig. 2E and F).

Next, it was found that the pretreatment of HUVECs with ZCZ significantly protected MMP from the negative effects of H_2_O_2_ (Fig. 2G). Compared with the H_2_O_2_ group (1.32), the green-red fluorescence ratio in ZIF-8, ZnO_2_, and CeO_2_ groups all decreased to varying degrees, being 1.12, 1.09 and 0.94, respectively. After pretreatment with Z_1_C_1_Z and Z_1_C_2_Z, the green fluorescence/red ratio further decreased to 0.83 and 0.54, respectively, indicating increased MMP (Fig. 2G–S15).

Cellular DNA damage is also a result of excessive oxidative stress, and γ-H2AX serves as a biomarker that can clearly reflect the degree of DNA damage and repair. As shown in Fig. 2H and I, the addition of ZCZ significantly reduced the expression of γ-H2AX in the nucleus compared with the H_2_O_2_ control group, suggesting a good protective function against DNA damage. These results collectively confirmed the protecting effect of ZCZ to HUVECs from oxidative stress damage. The above evidence indicates that ZCZ, as a nano-composite, has a stronger ability to resist oxidative stress damage compared with ZIF-8, ZnO_2_ and CeO_2_ alone.

### 3.5 ZCZ promotes the polarization of macrophages from M1 to M2

The repair process of skin injury includes multiple phases of coagulation, inflammation, proliferation and remodeling. Among these phases, two transition process including the polarization of macrophages and neovascularization are significant to wound healing [43]. As for normal wounds, the moderate inflammation is positive for pathogen removal, and the inflammatory phase enters the repair phase smoothly within 3-10 days [2]. However, in diabetic wound, a prolonged inflammatory response prevents the onset of the proliferative and remodeling phases [1,44]. In addition to the aggravation of oxidative stress damage, another major reason for the prolonged inflammatory phase is the abnormal polarization of macrophages [45]. In brief, impaired polarization of M1 to M2 allows M1 to persist, resulting in the accumulation of inflammatory cytokines and the arrest of the inflammatory phase rather than the failure to enter the repair phase [45].

**Fig. 3:**
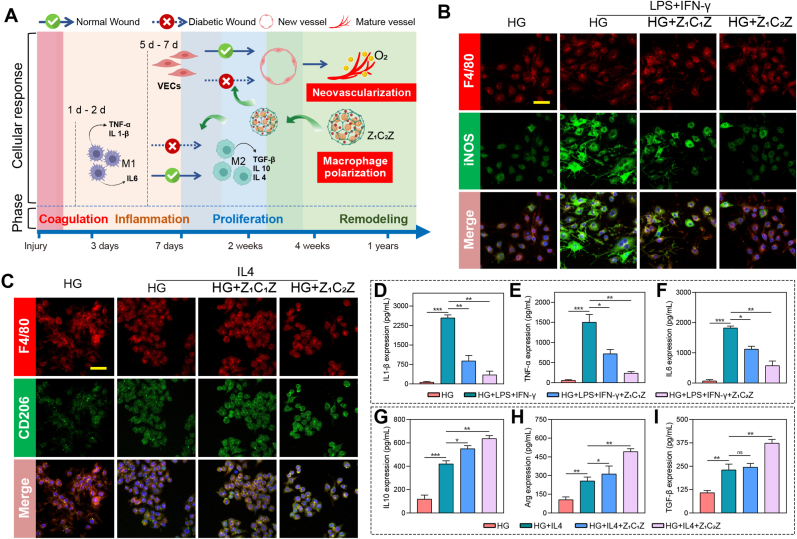
Effect of nano-materials on macrophage polarization (A) Illustration of the healing process compared between diabetic and normal wounds. Immunofluorescence images of the (B) M1 macrophage marker iNOS and (C) M2 macrophage marker CD206 in RAW 264.7 after different treatments. n = 3. Scale bar: 20 μm. (D-I) ELASA analysis of cytokine (M1: TNF-α, IL1-β and IL 6; M2: IL4, TGF-β and IL10) content secreted by RAW 264.7 cocultured with high glucose medium and three different nano-materials. n = 3. Data are mean standard error of the mean, p values are based Student's *t*-test. ∗p < 0.05, ∗∗p < 0.01, ∗∗∗p < 0.01, ns, nonsignificant.

In order to study the effect of ZCZ towards the polarization of macrophages, RAW 264.7 was pretreated with Z_1_C_1_Z and Z_1_C_2_Z, then M1 macrophage inducer (60 ng/mL LPS and 20 ng/mL INF-γ) and 30 mmol/L glucose were added to simulate inflammatory macrophages in HG environments. The results of immunofluorescence in Fig. 3B showed that LPS and IFN-γ induced the number of iNOS positive cells increased significantly. Moreover, the cells become elongated and irregular from a round-like shape, which is also an important feature of M1 macrophages. Interestingly, in the ZCZ pretreatment groups, the number of iNOS^+^ cells gradually decreased (Fig. 3B–S16). On the contrary, IL4 (20 ng/mL, M2 macrophage inducer) was used to induce polarization of RAW246.7 cells towards M2 macrophages, and the expression of CD206 was elevated. Then, the number of CD206^+^ cells in ZCZ group was gradually increased compared with that in IL4 only group (Fig. 3C–S17), suggesting that ZCZ induced macrophage polarization to M2. ELASA analysis was performed on the supernatant of RAW 264.7 culture, and it was found that ZCZ significantly decreased the concentration of pro-inflammatory cytokines secreted by M1, such as TNF-α, IL1β and IL6 (Fig. 3D–F), while the concentration of pro-repair cytokines secreted by M2, including arginase (Arg), TGF-β and IL10, increased (Fig. 3G–I).

### 3.6 ZCZ improves blood vessel function and promotes oxygen production in vitro

As was described in Fig. 3A, the obstruction of vascularization is another important reason for the delay of diabetic wound healing, which will lead to more severe ischemia and hypoxia in wound microenvironment [46,47]. Although there are many clinical treatments to improve vascular function, such as vascular interventional therapy, vascular stenting or balloon dilation can unclog blood vessels with a diameter of more than 2 mm, but it is almost useless for skin capillaries [41]. Therefore, it is still a practical clinical problem to change the microenvironment of hypoxia and ischemia in diabetic wounds.

**Fig. 4:**
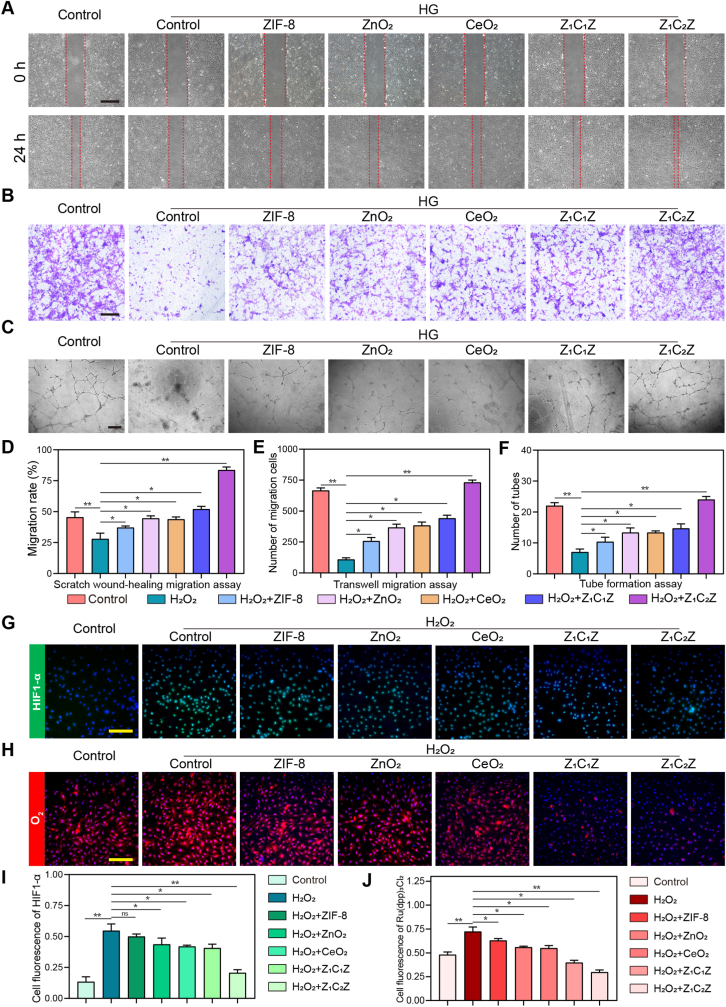
Nano-materials promoted the proliferation, migration, tube formation and oxygen production of HUVEC in the mimical diabetic wound microenvironment in vitro (A, B) Scratch wound-healing migration assay images of HUVECs treated with Z_1_C_1_Z and Z_1_C_2_Z (50 μg/mL) in high glucose medium for 24 h n = 3. Scale bar: 500 μm. (C, D) Typical images of transwell migration assays of HUVECs under different treatment. n = 3. Scale bar: 500 μm. (E, F) Image of tube formation of HUVECs in the presence of high glucose with or without nano-materials. n = 3. Scale bar: 500 μm. (G, H) Representative staining image of HIF-α expression in HUVECs cultured with Z_1_C_1_Z and Z_1_C_2_Z (50 μg/mL) after H_2_O_2_ (5 mM) pretreatment. n = 3. Scale bar: 200 μm. (I, J) Immunofluorescence images of HUVECs oxygen production with Ru(dpp)_3_Cl_2_ probes under different treatments. n = 3. Scale bar: 200 μm. Data are mean standard error of the mean, p values are based Student's *t*-test. ∗p < 0.05, ∗∗p < 0.01, ∗∗∗p < 0.01, ns, nonsignificant.

The effects of ZCZ on vascularization were tested mainly from proliferation, migration and tubeforming ability of HUVECs in HG environments. First, proliferation of HUVECs was decreased in the HG group compared to the normal glucose control group, while cell proliferation was significantly enhanced after Z_1_C_1_Z and Z_1_C_2_Z administration (Fig. S18). To determine the promoting effects of ZCZ on HUVECs migration under high glucose culture, serum-starved HUVECs were co-cultured with 50 μg/mL of Z_1_C_1_Z and Z_1_C_2_Z and high glucose medium for 24 h. Scratch wound-healing migration assay showed that Z_1_C_1_Z and Z_1_C_2_Z increased the migration rate of HUVECs from 28.0% to 62.9% and 83.5%, respectively, compared with the high glucose control group (Fig. 4A–D). Furthermore, transwell migration assays also suggested that the addition of ZCZ nano-materials increased the number of cells passing through the well compared with the high glucose culture alone (Fig. 4B–E). Finally, we evaluated the angiogenesis ability of HUVECs through tube formation assay. As shown in Fig. 4C and F, compared with the high glucose group, the number of HUVECs forming tubules in the Z_1_C_2_Z group increased from 7 to 15 per visual field, and the vessel circumference also increased by nearly 2 times.

In addition to improving vascular function, the oxygen supply is also beneficial in diabetic wounds healing. Clinically, hyperbaric oxygen therapy is a common certain option for patients with diabetic wounds, the wounds will return to the original hypoxia situation when patients leave the hyperbaric oxygen chamber. Therefore, we evaluated the effect of the prepared ZCZ nano-materials on oxygen supply. Firstly, H_2_O_2_ treatment increased HIF-1α expression in HUVECs compared with normal culture (Fig. 4G), suggesting that H_2_O_2_ treatment induces a hypoxic microenvironment. After treatment with ZIF-8, ZnO_2_, CeO_2_, Z_1_C_1_Z and Z_1_C_2_Z, the expression of HIF-1α was gradually decreased, indicating that the nano-materials may improve their hypoxic distress (Fig. 4G and I). For a more direct observation of oxygen generation, Ru(dpp)_3_Cl_2_, a widely used fluorescent probe for the detection and quantification of oxygen, was used to label HUVECs. The fluorescence of Ru(dpp)_3_Cl_2_ will be quenched by oxygen, it can be found that ZIF-8, ZnO_2_, CeO_2_ alone and ZCZ nano-composite all have the ability of oxygen supply, among which Z_1_C_2_Z is the strongest (Fig. 4H–J).

In short, the ZCZ nano-composite can promote neovascularization of HUVECs through enhancing its proliferation, migration and tube formation under high glucose environment, and provided continuous oxygen generation, which well improved the ischemia and hypoxia microenvironment of diabetic wounds.

### 3.7 ZCZ promotes wound healing of diabetic mice in vivo

**Fig. 5:**
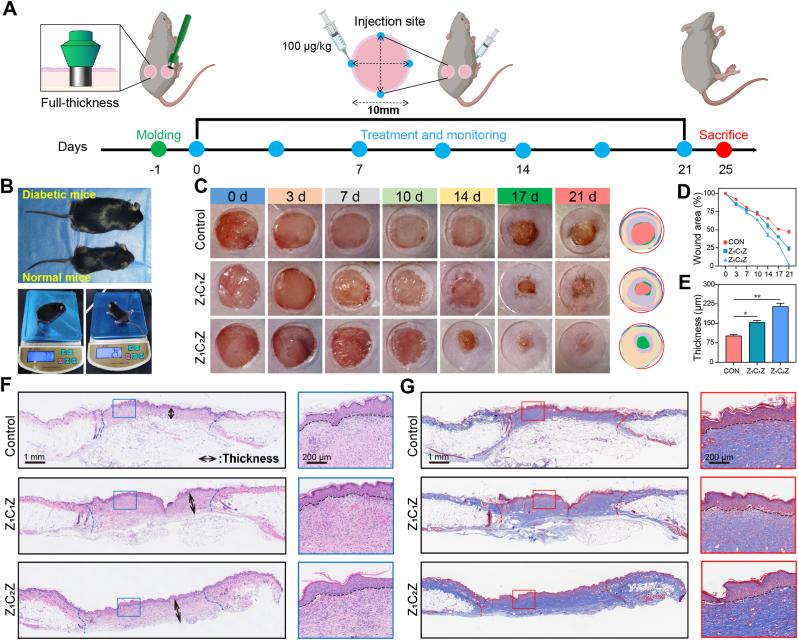
Evaluation of wound healing in different groups of diabetic mice (A) Schematic illustration of the experimental design. (B) Images of body size and weight comparison between normal (C57) and diabetic (db/db) mice. (C) Photographs of diabetic mice models treated with Z_1_C_1_Z, and Z_1_C_2_Z at different time points within 3 weeks. n = 3. (D) Quantitative analysis of the percentage of residual wound during 3 weeks of different treatments. (F) Representative HE staining images of wound healing after different treatments for 3weeks. n = 3. (E) Quantitative analysis of wound dermal thickness of diabetic mice in different treatment groups. (G) Representative masson's trichrome staining images of wound healing after different treatments for 3 weeks. n = 3. Data are mean standard error of the mean, p values are based Student's *t*-test. ∗p < 0.05, ∗∗p < 0.01, ∗∗∗p < 0.01, ns, nonsignificant.

The wound healing experiments were illustrated in Fig. 5A, a hole punch was used to create two round, full-thickness skin wounds with a diameter of 10 mm on the back of the mice. Next, a solution of 0.1 mL ZCZ material (100 μg/kg) was injected into each of the four vertices of the wound every 7 days, while the wound healing was followed up. On day 25, the mice were euthanized and skin tissues were sent for pathological staining. Firstly, C57 mice were utilized to investigated the effect of ZCZ on normal wound. On the first day after modeling, the solution of ZCZ at the dosage of 100 μg/kg were injected into the wound edge, and the control group was injected with PBS. Weight, blood glucose, and wound healing related indicators were followed up after surgery. In Fig. S19A–C, our follow-up suggested that the unhealed wound area of Z_1_C_1_Z and Z_1_C_2_Z groups on day 14 was 0.210 cm^2^ and 0.129 cm^2^, respectively, which was significantly less than that of the control group (0.304 cm^2^). Further HE staining also suggested that the treatment group showed better healing quality, the wound dermis in group Z_1_C_2_Z was the thickest (Fig. S20A and B). Collagen, as a major component of the extracellular matrix, is an important indicator of wound healing. Masson's trichrome staining of the tissue was performed, blue represents collagen, while other tissue or cellular components (cell bodies, muscle, and keratin, among others) were stained red. Fig. S21A and B suggested that the proportion of collagen in the Z_1_C_2_Z group was the highest. In addition, the content of hydroxyproline, one of the main components and unique amino acids of collagen, was measured to be 3.3 mg/g, while those in Z_1_ZC_1_and Z_1_C_2_Z groups were 5.2 mg/g and 7.7 mg/g, respectively (Fig. S22).

To further determine the effectiveness on wound healing in diabetic mice, db/db mice, a well-established diabetic mouse model, were selected for in vivo experiments. Compared with normal C57BL mice, db/db mice have a higher blood glucose (8.7-12.3 mmol/L) and a larger body weight (36-43 g) (Fig. 5B–S12, S23). Using a similar modeling method, as shown in Fig. 5A, we created 2 skin wounds on the back of db/db mice. On the first day after modeling, the mice were randomly divided into three groups: Z_1_C_1_Z and Z_1_C_2_Z groups were injected with 100 μg/kg of drug around the wound, respectively, while the control group was injected with the same volume of PBS. The weight of db/db mice only decreased slightly within 3-10 days after the operation, which may be caused by the surgical stimulation response (Fig. 5B–S12). On the 21st day, the unhealed wound area of control, Z_1_C_1_Z and Z_1_C_2_Z groups were 0.315, 0.235, and 0.028 cm^2^ (Fig. S24), and the proportion of unhealed wound area was 47.3%, 34.0% and 2% respectively (Fig. 5D). After sampling the completely healed wound skin, HE staining showed that the wound healing quality of Z_1_C_2_Z group was significantly better than that of the control group (Fig. 5F). The average thickness of dermal tissue in Z_1_C_1_Z and Z_1_C_2_Z groups was about 1.5, and 2.1 times that of the control group, respectively (Fig. 5E). Masson staining results showed that the average proportion of collagen in the control, Z_1_C_1_Zand Z_1_C_2_Z groups was 40.0%, 61.6% and 61.3% (Fig. 5G–S25). At the same time, the Z_1_C_2_Z group had the highest hydroxyproline value (Fig. S26), which indicated that the nano-materials promoted the accumulation of collagen in wound. The experiments in normal and diabetic mice collectively showed remarkable ability of ZCZ nano-materials to promote wound healing, especially in db/db mice.

### 3.8 ZCZ reduces inflammation and promotes neovascularization in vivo

**Fig. 6:**
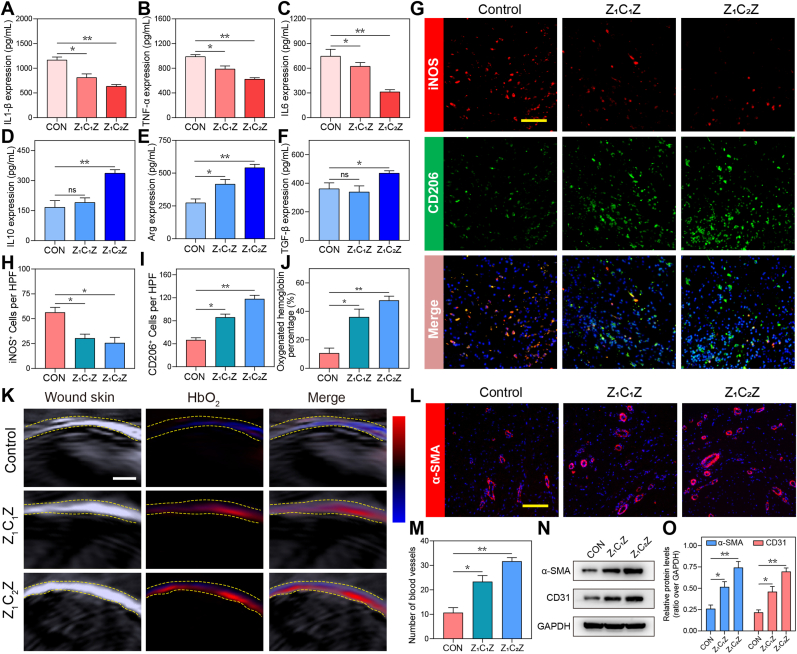
Effects of different nano-materials on angiogenesis, inflammation, and oxygen production in diabeti wound (A-F) Quantitative analysis of TNF-α, IL1-β, IL6, Arg, TGF-β and IL10 content in different groups of wounds by ELASA assay. n = 3. (G, H, I) Fluorescent staining images of infiltration of two types of macrophages labeled with iNOS (M1) and CD206 (M2) in diabetic wounds after modeling 2 weeks. n = 3. Scale bar: 200 μm. (J) Statistical analysis of oxygenated hemoglobin in photoacoustic detection. (K) Photoacoustic images of db/db mice wounds after treatment with Z_1_C_1_Z, and Z_1_C_2_Z. n = 3. Scale bar: 1 mm. (L) Representative fluorescence images of blood vessels (marked with α-SMA protein) after wound healing in different groups. Scale bar: 500 μm. (M) Statistical analysis of the number of vessels in each field of view. (N) Western Blot and (O) statistical analysis of α-SMA and CD31 in different groups. n = 3. Data are mean standard error of the mean, p values are based Student's *t*-test. ∗p < 0.05, ∗∗p < 0.01, ∗∗∗p < 0.01, ns, nonsignificant.

The transition stage from inflammatory phase to proliferative phase of wound healing is about 14 days. During this period, macrophages in the tissue are polarized from M1 to M2, thereby promoting wound repair. Therefore, the samples were taken from the wounds 2 weeks after modeling. First, the ELISA assay showed the levels of TNF-α, IL1β and IL6 in the wound tissue of Z_1_C_1_Z and Z_1_C_2_Z groups were decreased, while the levels of Arg, TGF-β and IL10 were visibly increased compared with the control group (Fig. 6A–F). To facilitate a more intuitively observation of macrophage polarization in vivo, the skin wound tissue was detected by immunofluorescence. In Fig. 6G, H and I, the images indicated that the number of iNOS^+^ cells was the highest in the control group, while the number of CD206^+^ cells was the highest in the Z_1_C_2_Z group, indicating that the nano-composite promoted the polarization of macrophages from M1 to M2. These results all imply that our nano-materials have potent anti-inflammatory effects and promote macrophage polarization to M2.

The wound generally enters the proliferative phase approximately two weeks post-injury. Neovascularization, an important indicator during this period, could give more oxygen supply and better tissue growth. So, the function of these neovascularization in the wound was examined by photoacoustic imaging. Compared with the control group, the Oxygenated Hemoglobin content of Z_1_CZ_1_, and Z_1_C_2_Z groups increased, among which Z_1_C_2_Z was the most obvious, suggesting that it is a good “oxygen pump” (Fig. 6J and K). Immunofluorescence staining of α-SMA, a marker of mature blood vessels, was performed on the completely healed wound. The number of blood vessels in the Z_1_C_2_Z group was the largest, about 1.86 times that of the control group (Fig. 6L and M). In addition, Western Blot analysis revealed that ZCZ treatment upregulated the expression of CD31 and α-SMA protein (two markers of angiogenesis) in different experimental groups (Fig. 6M–O).

### 3.9 ZCZ exerts its effects through the IL-6/JAK2/STAT3 signaling pathway

**Fig. 7:**
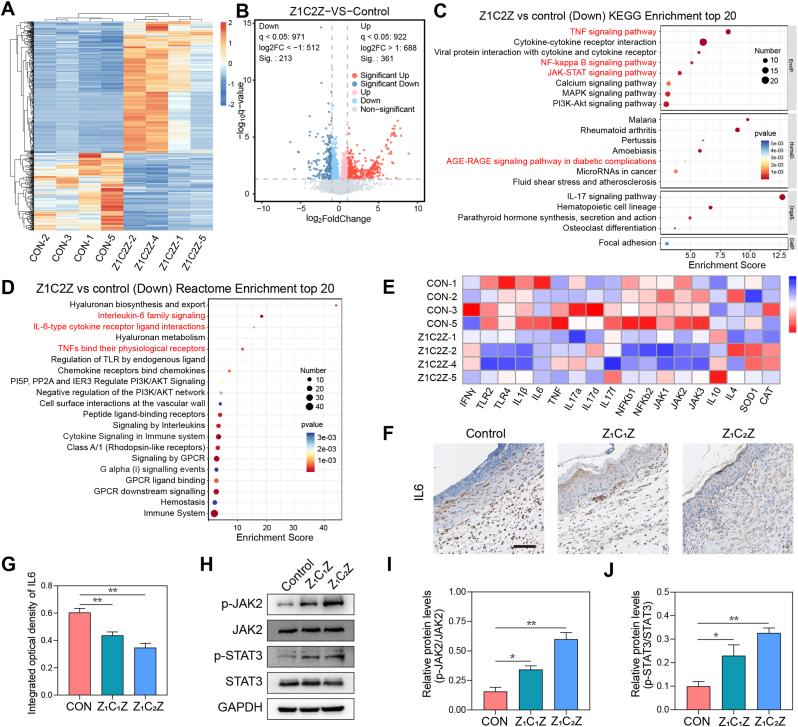
Transcriptome sequencing for investigating potential mechanisms of ZCZ therapy (A) Heatmap displayed the differentially expressed genes (DEGs) in control and ZCZ groups. The ordinate is the value after the normalization of Fragments Per Kilobase of exon model per Million mapped fragments (FPKM), and the more orange the color, the higher the expression. (B) Volcano map showed the top differentially expressed genes (DEGs) in control and ZCZ groups. Red means up-regulated, blue means down-regulated. (C) Reactome pathway enrichment analysis revealed a significant down-regulation of gene-associated signaling pathways in the top 20. (D) The KEGG pathway enrichment analysis revealed a significant down-regulation of gene-associated signaling pathways in the top 20. (E)Hot maps of gene expression representative of precursors to inflammation and oxidative stress. (F) Immunohistochemical staining images and (G) statistical analysis of IL6. n = 3. Scale bar: 100 μm. (H) Western Blot and (I, J) statistical analysis of p-JAK2, JAK2, p-STAT3 and STAT3. (J) The illustrative image of the IL6/JAK2/STAT3 signaling pathway. Data are mean standard error of the mean, p values are based Student's *t*-test. ∗p < 0.05, ∗∗p < 0.01, ∗∗∗p < 0.01, ns, nonsignificant. (For interpretation of the references to color in this figure legend, the reader is referred to the Web version of this article.)

To further investigate the potential anti-inflammatory mechanism of ZCZ, transcriptome sequencing was performed on wound skin subsequently. Compared with the control group, 361 genes were significant up-regulated and 213 genes were down-regulated in the skin wound tissue of ZCZ-treated mice, as shown in the heat and volcano maps in Fig. 7A and B. KEGG pathway enrichment analysis showed that ZCZ-induced differentially expressed genes (DEGs) was correlated with “TNF signaling pathway”, “NF-kappa B signaling pathway”, “JAK-STAT signaling pathway” and “AGE-RAGE signaling pathway in diabetic complications” (Fig. 7C). Reactome pathway enrichment analysis suggested that IL6 signaling pathway may play an important role (Fig. 7D). Indeed, IL6-mediated JAK2/STAT3 signaling pathway exerts an classic influence on the process of macrophage polarization. Further, heat map in Fig. 7E depict the expression patterns of DEGs associated with inflammation and oxidative stress. Expressions of M1 macrophage-associated inflammatory factors INFγ, IL1β, IL6, and TNF were elevated in the ZCZ group, while expressions of M2 macrophage-associated cytokines such as IL4 and IL10 were low, compared with the control group (red for high expression, blue for low expression, Fig. 7E). Those sequencing data were consistent with the findings from in vitro experiments, suggesting that ZCZ also exhibited anti-inflammatory effects and promoted M2 macrophage polarization in vivo. The mechanistic action of ZCZ may involve the IL6/JAK2/STAT3 signaling pathway. Mechanically, ZCZ may function primarily through the IL6/JAK2/STAT3 signal axis. Therefore, we examined the protein levels associated with this signaling pathway. The polarization direction of macrophages (M1 or M2) is closely related to the selective gene expression regulation mediated by different signaling pathways of inflammatory factors [48]. The IL6/JAK/STAT3 signaling pathway has been verified as a crucial determinant in regulating the polarization of macrophages towards the M2 type [49]. The specific mechanism is as follows [[49], [50], [51]]: (1) The cytokine IL6 binds to the IL 6R on the surface of macrophage, activates GP130, and prompts it to form a homodimer; (2) The JAKs (JAK1/2) associated with the receptor draw closer to each other and establish corresponding STAT3 docking sites via mutual phosphorylation; (3) The activated JAKs recruit STAT3 molecules that exist as monomers in the cytoplasm; (4) The phosphorylated STAT3 molecules form homodimers or heterodimers and are released from the receptor, entering the nucleus in dimer form to initiate the transcription of related genes and induce the polarization of macrophages towards the M2 type. In Fig. 7F and G, the expression of IL6 in the control group was significantly higher than that in the ZCZ group, showing that ZCZ treatment reduced the inflammatory response. Next, we observed the expression of phosphorylated JAK2 (p-JAK2) and STAT3 (p-STAT3), and noted that ZCZ treatment significantly augmented the levels of p-JAK2 and p-STAT3, indicating a modulatory effect of ZCZ on the JAK2/STAT3 signaling pathway (Fig. 7H–J).

The IL6/JAK/STAT signaling pathway has been demonstrated to exert an impact on wound healing through the regulation of macrophage polarization and angiogenesis [52,53]. Inhibition of NF-κB/JAK-STAT signaling pathway could promote the healing of diabetic wounds [54]. Our study showed the efficacy of ZCZ in effectively inhibiting IL6/JAK2/STAT3 signaling, thereby promoting diabetic wound healing. However, our mechanism studies have limitations, such as the absence of experiments targeting the IL6/JAK2/STAT3 pathway using inhibitors or genetic interventions. In the future, more experiments will be conducted to thoroughly understand the complex regulation of the IL6/JAK2/STAT3 pathway by ZCZ.

## 4 Discussion

The primary factor contributing to impaired healing in diabetic wounds is the pathological microenvironment induced by hyperglycemia, which leads to a significant increase in free radicals (e.g., ROS) and a concurrent reduction in the body's antioxidant defense capacity [55]. This imbalance results in oxidative stress (OS), causing tissue damage that is specifically manifested as inadequate blood oxygen supply and an excessive inflammatory response [56]. A key contributing factor is the dysregulated macrophage polarization process, characterized by a failure to transition from the pro-inflammatory M1 phenotype to the anti-inflammatory M2 phenotype [44,57]. This disruption sustains a persistent pro-inflammatory state and establishes a positive feedback loop that amplifies inflammation [58]. Concurrently, it leads to diminished cytokine production and impaired vascularization, which collectively represent central mechanisms underlying the majority of pathological alterations observed in diabetic wounds [58]. Therefore, targeting ROS scavenging and enhancing oxygen supply to promote M2 macrophage polarization and angiogenesis has emerged as a key therapeutic strategy for the treatment of diabetic wounds.

In the last few years, MOFs have demonstrated considerable potential in the therapeutic application for diabetic wounds, owing to their high drug loading capacity, biodegradability, and stimuli-responsive drug release characteristics [59,60]. In particular, hybrid MOFs that integrate nanozymes with MOF structures offer a multifunctional platform for diabetic wounds and demonstrate significant therapeutic potential [22,61]. Nanozymes, a type of nano-material with highly efficient catalytic activity similar to that of biological enzymes (such as oxidases and peroxidases), have been extensively utilized in the generation and scavenging of ROS [62,63]. CeO_2_ nanozymes can promote wound healing by exhibiting superoxide dismutase- and catalase-like activities, which effectively eliminate excessive ROS [64,65]. In our present study, CeO_2_ also played the role of oxidases and peroxidases, effectively catalyzing the conversion of ROS into O_2_. This represents a dual-functional strategy that concurrently facilitates the clearance of ROS and promotes endogenous O_2_ regeneration. Zinc ions have been clinically proven to promote wound healing [66,67]. Among them, zinc oxide is widely used, and zinc oxide NPs has also been applied [68]. However, compared to zinc oxide, ZnO_2_ carries O_2_^2−^, which can react with water to produce O_2_. Zinc ions are highly versatile and contribute to tissue repair through multiple mechanisms. Zinc ions that are continuously released from zinc-imbued metal-organic frameworks can promote bone repair by enhancing collagen regeneration and reducing inflammation [69]. Zinc ions (Zn^2+^) action could promote macrophage polarization from M1 to M2 phenotype, thereby releasing cytokines conducive to tissue regeneration [70]. The increase in the intracellular zinc ion concentration can effectively elevate the phosphorylation level of STAT3, thus promoting the expression of downstream genes such as α-SMA and COL1, and further facilitating the healing of skin wounds [71]. ZIF-8 enhanced the antibacterial activity of chitosan by releasing antibacterial Zn^2+^in a pH-responsive and sustainable manner, thereby promoting the healing of diabetic wounds [72].

Given the biological significance of zinc ions and the oxygen-supplying capacity of O_2_^2−^, we integrated component ZnO_2_ into our system.

In summary, we have successfully synthesized a nanocomposite ZCZ which showed significant efficacy in promoting diabetic wound healing. The ZCZ possessed multifunctional properties including anti-oxidative stress, anti-inflammatory effects, continuous oxygen generation, and promotion of angiogenesis. It effectively eliminates excessive ROS from diabetic wounds and reduces oxidative stress levels. During the inflammatory response stage, it facilitates the polarization of macrophages from M1 to M2 phenotype via IL6/JAK2/STAT3 signaling pathway, thereby expediting the transition to the proliferative repair phase. In the repairing stage, our developed ZCZ remarkably enhanced angiogenesis and stimulated oxygen production within diabetic wounds, consequently ameliorated ischemic and hypoxic conditions and accelerated wound healing process. Intriguingly observations in vivo experiments demonstrated that ZCZ exhibited effectiveness not only in diabetic wounds but also in normal wounds, suggesting its broad application potential as a novel therapeutic strategy for non-diabetic wounds.

## Ethics approval and consent to participate

This study was approved by the Ethics Committee for Clinical Research and Animal Trials of the First Affiliated Hospital of Sun Yat-sen University (Guangzhou, China, approval number: lunshen [2021] 168).

## CRediT authorship contribution statement

**Yunxian Dong:** Conceptualization, Funding acquisition, Methodology, Project administration, Writing – original draft, Writing – review & editing. **Lei Ren:** Data curation, Methodology, Project administration, Writing – original draft. **Xiaoling Cao:** Data curation, Investigation, Methodology, Software. **Zhongye Xu:** Methodology, Software. **Zhongping Zhang:** Methodology, Software, Validation. **Jian Wang:** Investigation, Methodology. **Shiqi Wang:** Software, Writing – review & editing. **Zirui Zhao:** Methodology, Software. **Dongming Lv:** Methodology, Writing – review & editing. **Yongqing Li:** Validation, Writing – review & editing. **Hui Fu:** Software, Writing – review & editing. **Zhigang Meng:** Methodology, Writing – review & editing. **Jia Tao:** Project administration. **Peng Zhao:** Conceptualization, Data curation, Project administration, Writing – original draft, Writing – review & editing. **Bing Tang:** Conceptualization, Formal analysis, Funding acquisition, Methodology, Project administration, Writing – original draft, Writing – review & editing. **Qing Tang:** Conceptualization, Formal analysis, Methodology, Project administration, Visualization, Writing – original draft.

## Declaration of competing interest

The authors declare that they have no known competing financial interests or personal relationships that could have appeared to influence the work reported in this paper.

## Data Availability

Data will be made available on request.
