# Supplementary material for: Metal organic framework based synergistic improvement of hypoxia for optimizing diabetic wounds healing
Source: Mater Today Bio. 2026 Apr 30;38:103169. doi: 10.1016/j.mtbio.2026.103169 (PMC13158423; doi:10.1016/j.mtbio.2026.103169)
Supplement: Multimedia component 1 [file mmc1.docx]

**Supplementary information**

**Synergistic Improvement of Hypoxia via Enhancing Exogenous and Endogenous Oxygen Supply for Optimizing Diabetes Wounds Healing**

Yunxian Dong^1, 2, #^, Lei Ren^1, #^, Xiaoling Cao^1, #^, Zhongye Xu^3,#^, Zhongping Zhang^1^, Jian Wang^1^, Shiqi Wang^1^, Zirui Zhao^1^, Dongming Lv^1^, Yongqing Li^2^, Hui Fu^2^, Zhigang Meng^2^, Jia Tao^4^, Peng Zhao^5, *^, Bing Tang^1, *^, Qing Tang^1, *^

1 Department of Plastic Surgery, the First Affiliated Hospital of Sun Yat-sen University, Guangzhou 510080, China.

2 Department of Breast Surgery, Shandong Cancer Hospital and Institute, Shandong First Medical University and Shandong Academy of Medical Sciences, Jinan 250117, China.

3 Department of Burns, Wound Repair and Reconstruction, the First Affiliated Hospital of Sun Yat-sen University, Guangzhou 510080, China.

4 School of Chemistry and Chemical Engineering, South China University of Technology,Guangzhou 510640, China.

5 Guangdong Provincial Key Laboratory of New Drug Screening, School of Pharmaceutical Sciences, Southern Medical University，Guangzhou 510515, China.

**Supplementary Figure**

**
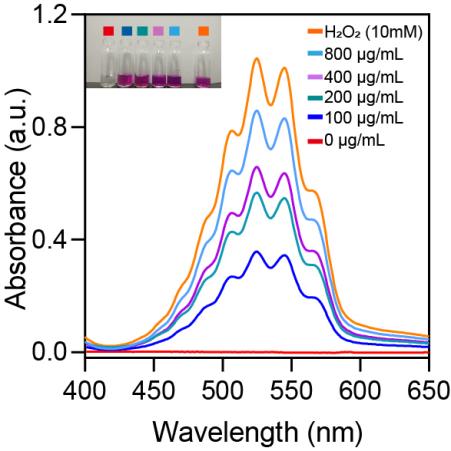
**

**Figure S1** The ability of ZnO_2_ to produce H_2_O_2_.

**
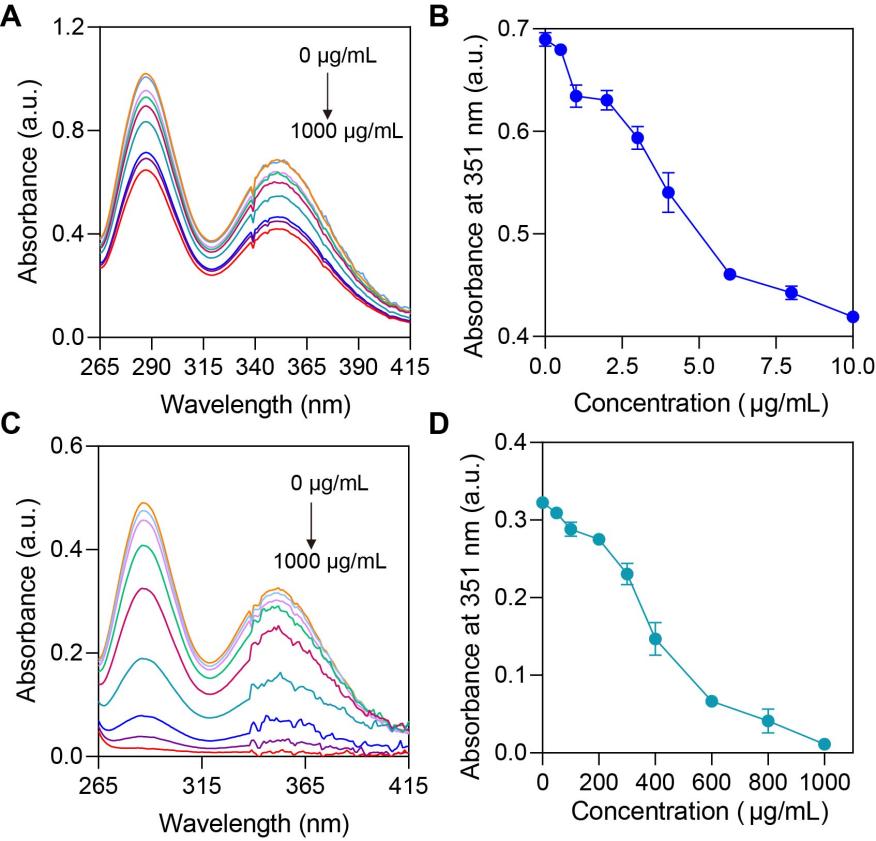
**

**Figure S2** The CAT-like activity of (A) CeO_2_ (0-1000 μg/mL) and the absorption peak value at 351 nm. The CAT-like activity of (C) ZCZ (0-1000 μg/mL) and the absorption peak value at 351 nm.


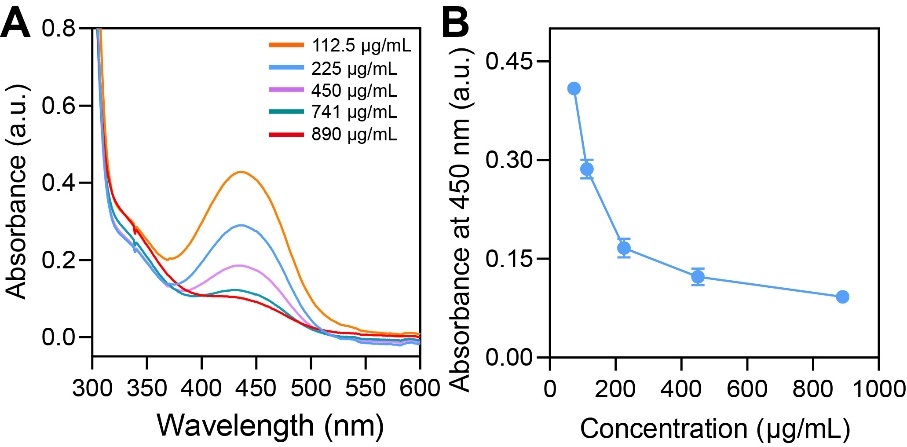


**Figure S3** (A)The SOD-like activity of ZCZ with different concentrations and (B)the absorption peak value at 450 nm.

**
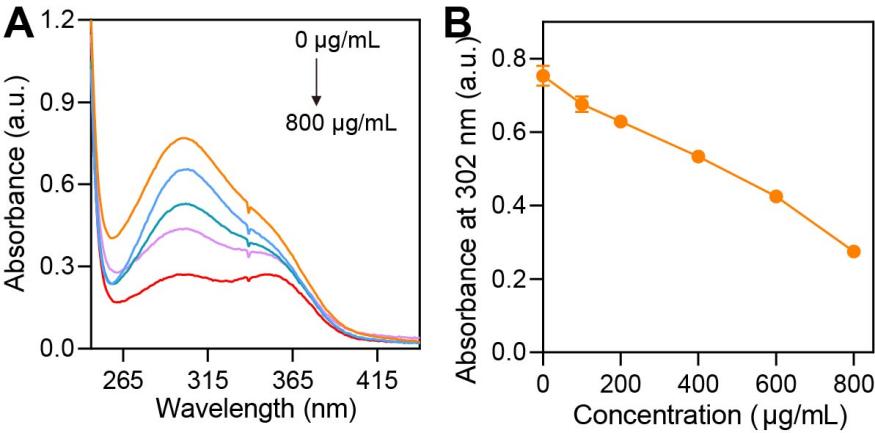
**

**Figure S4** (A)The capacity of ZCZ for the removal of reactive nitrogen species (RNS) including ONOO^-^ and (B) the absorption peak value at 302 nm.


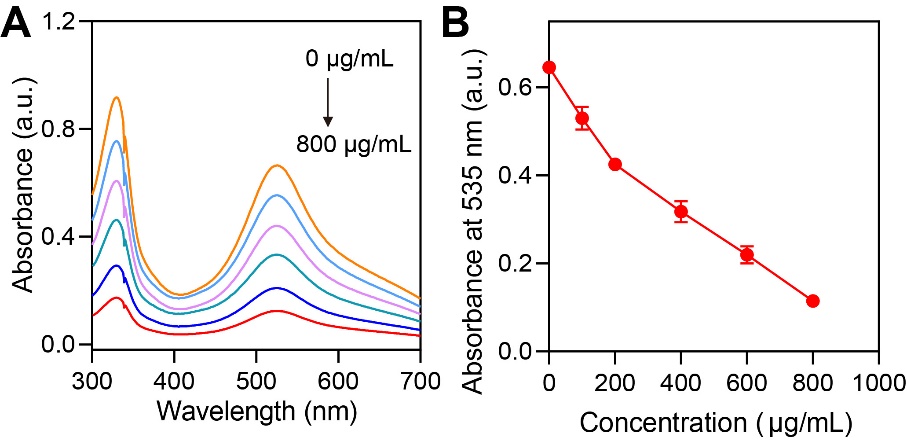


**Figure S5** (A)The scavenging ability of ZCZ against DPPH and the absorption peak value at 535 nm.

**
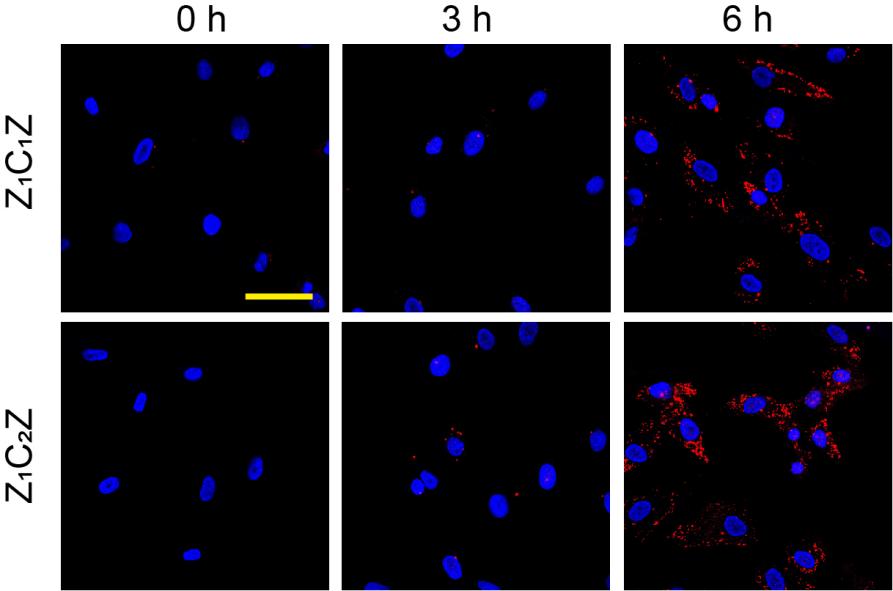
**

**Figure S6** Stained images demonstrating celler uptake of the three nanodrugs in HUVECs for 0-6 h. Scale bar: =20 µm.


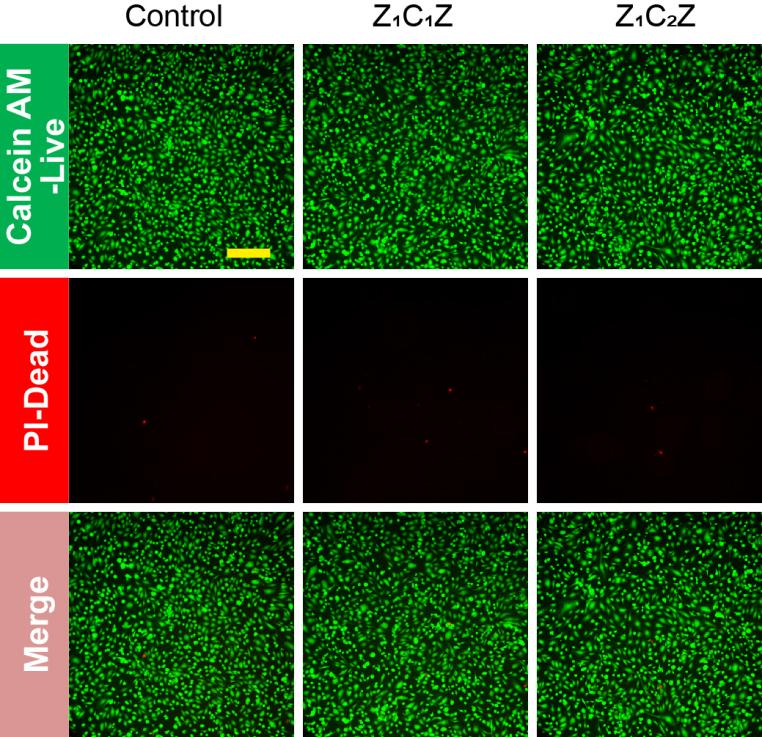


**Figure S7** Representative images of live (calcein, 2µM, green) or dead (propidium iodide, 8µM, red) HUVECs under different treatments. n=3. Scale bar: 200 µm.


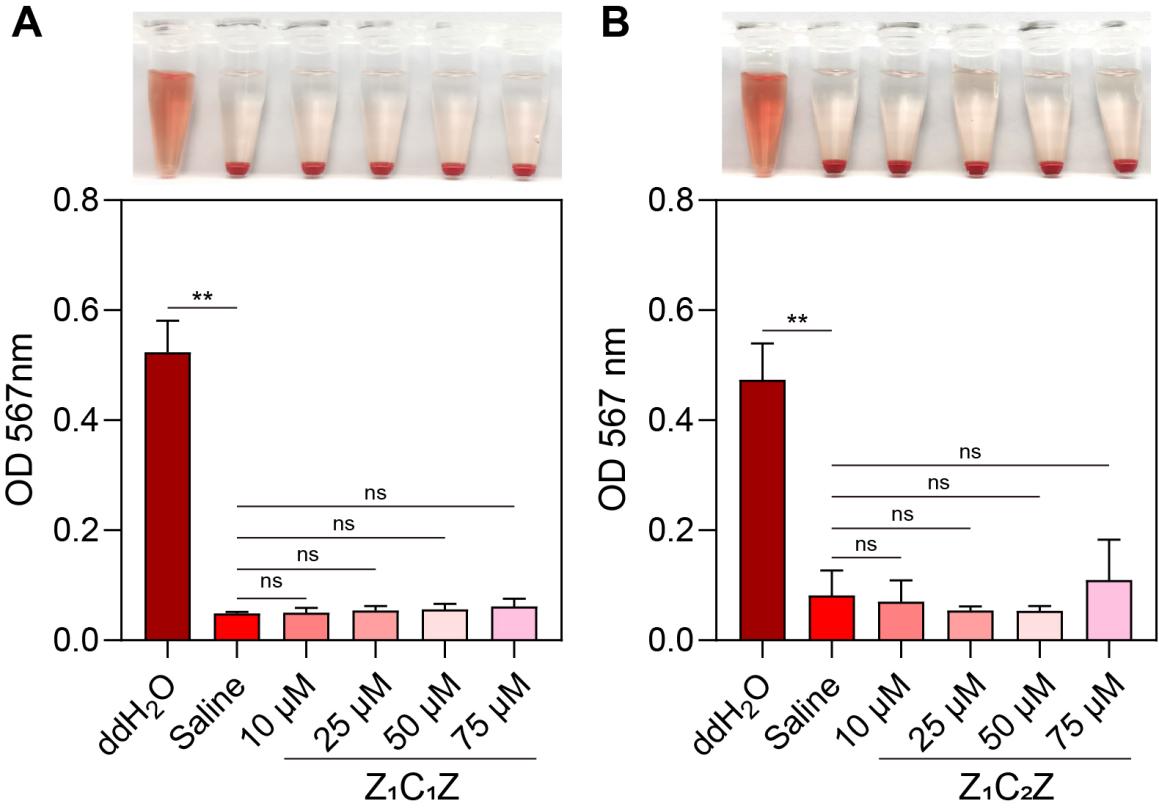


**Figure S8** Hemolysis test images of (A) Z_1_C_1_Z and (A) Z_1_C_2_Z with fresh C57 mouse erythrocytes. Quantitative analysis was performed by measuring the optical density of the sample at OD_567nm_. *p < 0.05, **p < 0.01, ***p < 0.01, ns, nonsignificant.


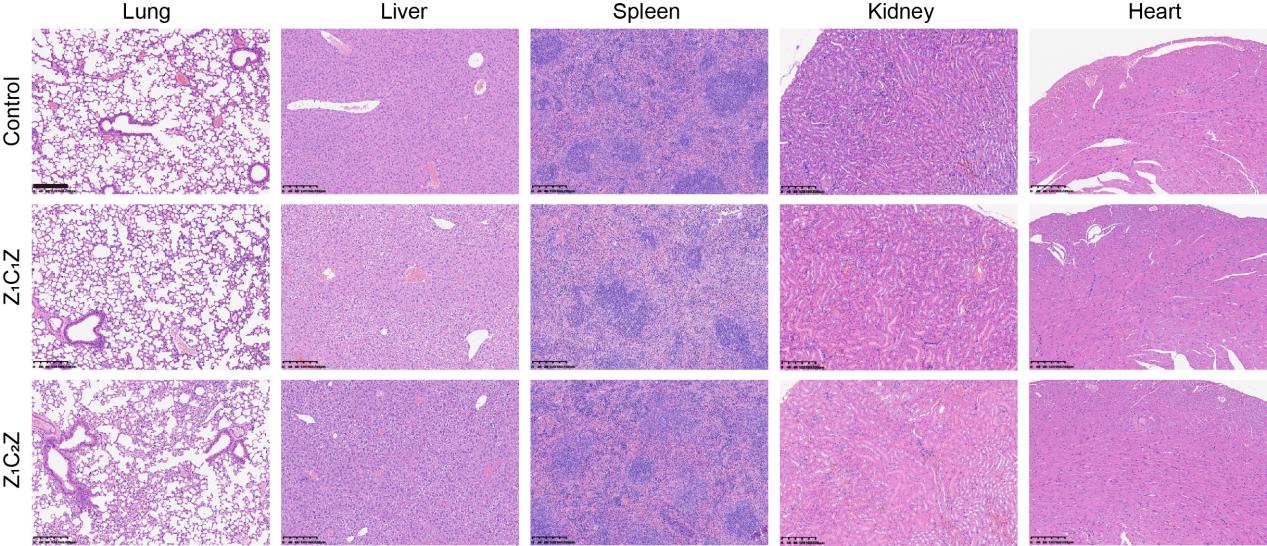


**Figure S9** HE staining of heart, liver, lung, spleen and kidney 14 days after different treatment with C57 mice. n=3. Scale bar: =200 µm.


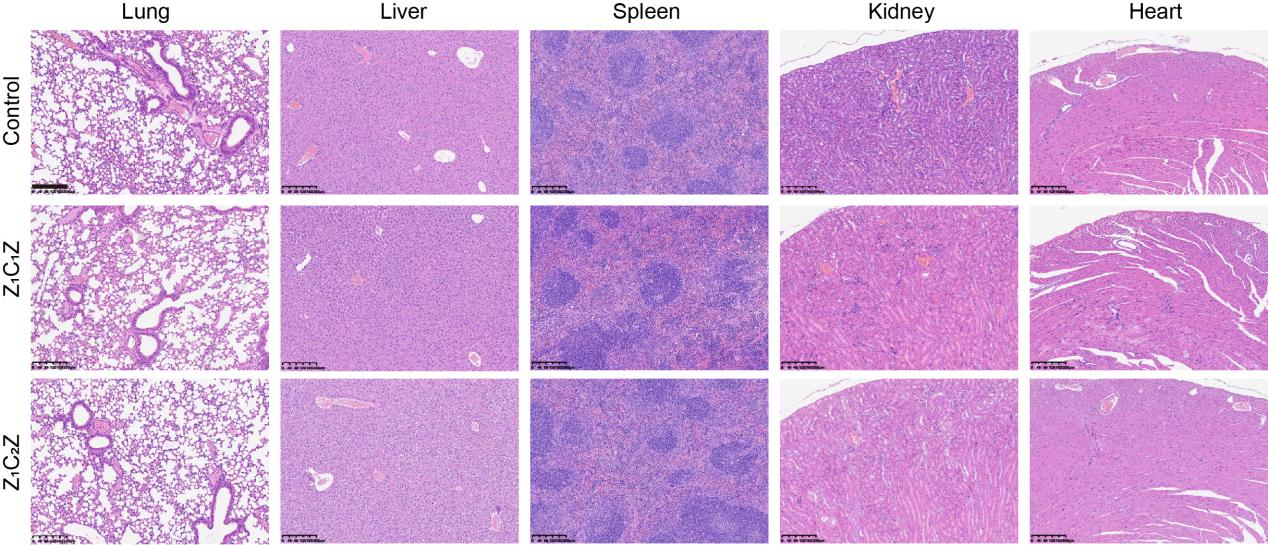


**Figure S10** HE staining of heart, liver, lung, spleen and kidney 14 days after different treatment with db/db diabetic mice. n=3. Scale bar: =200 µm.


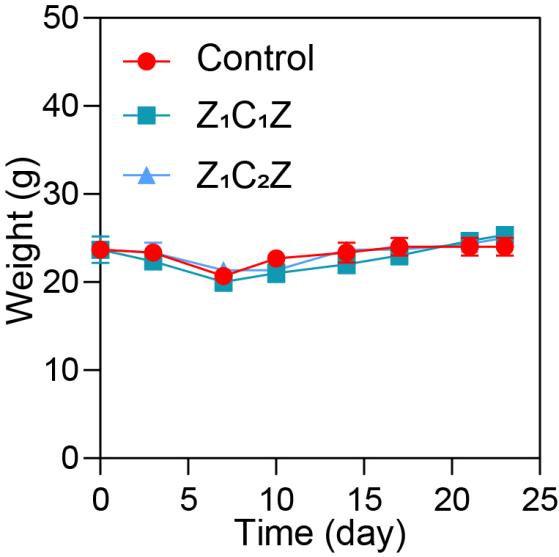


**Figure S11** Changes in body weight of C57 mice after Z1C1Z and Z1C2Z treatment.


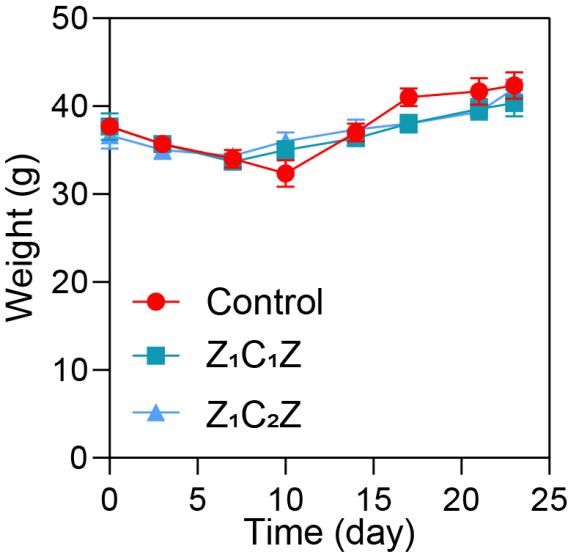


**Figure S12** Changes in weight of diabetic mice (db/db) after Z1C1Z, and Z1C2Z treatment.


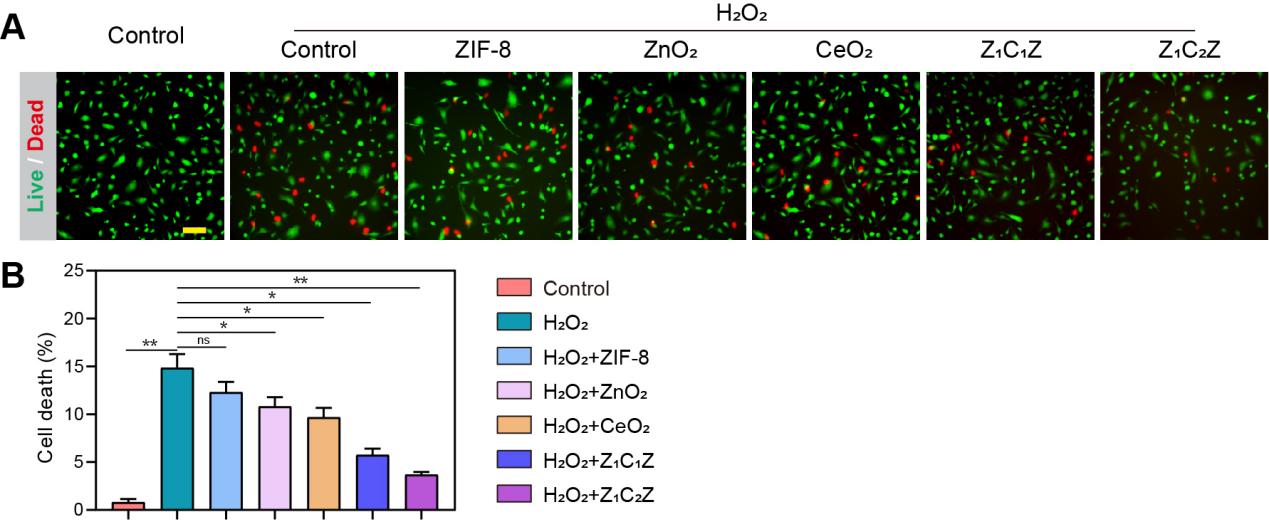


**Figure S13** Live/dead fluorescent images of HUVECs incubated with Z_1_C_1_Z and Z_1_C_2_Z (50 µg/mL) with or without H_2_O_2_ (5 mM). n=3. Scale bar: 100 µm. *p < 0.05, **p < 0.01, ***p < 0.01.


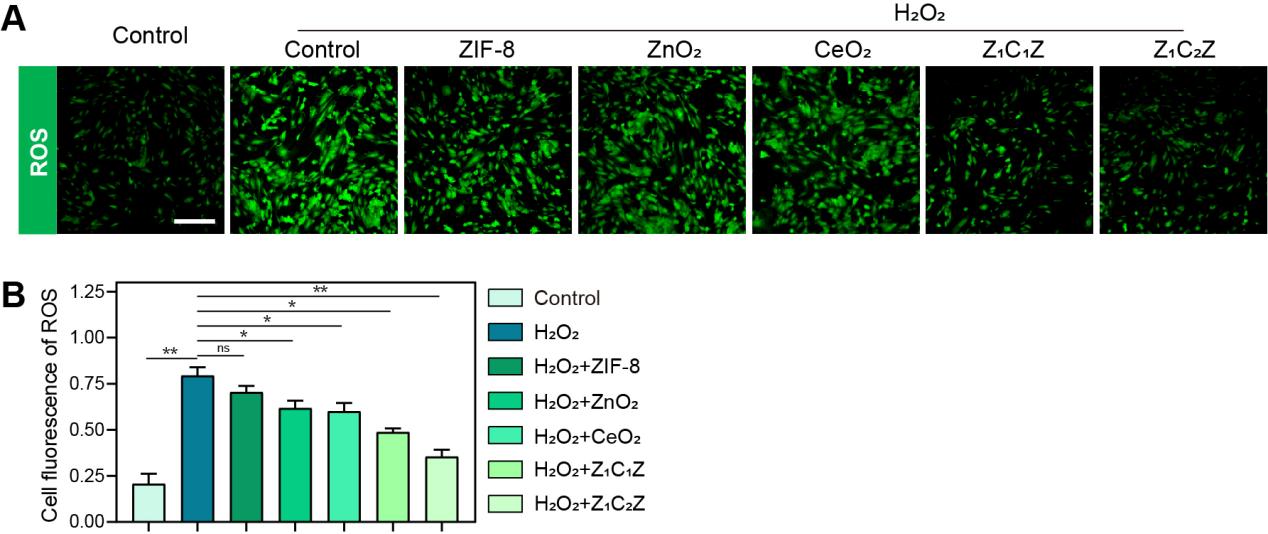


**Figure S14** Immunofluorescence images of DCFH-DA showed the effect of different drugs on ROS scavenging, n=3. Scale bar: 100 µm. *p < 0.05, **p < 0.01, ***p < 0.01.


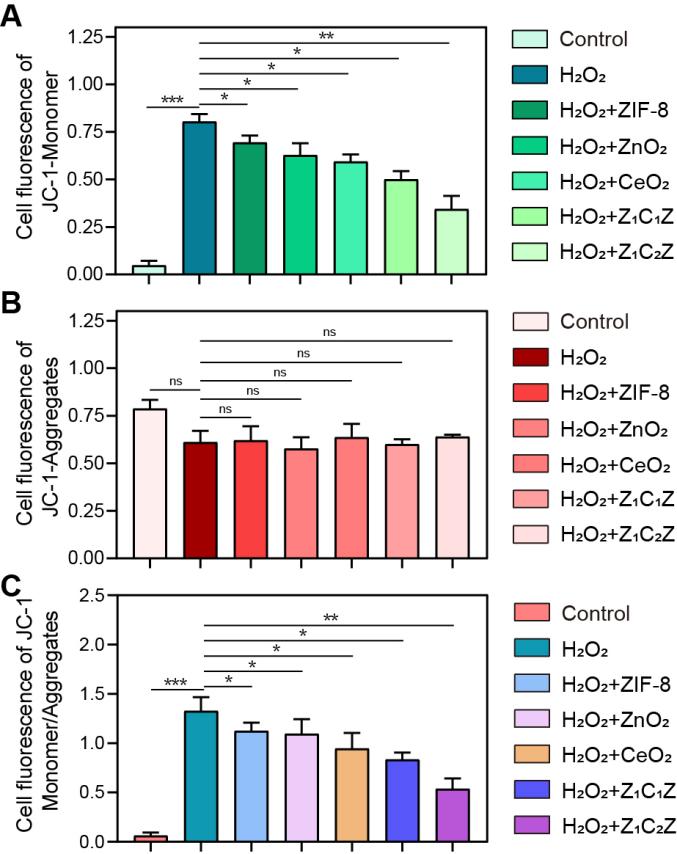


**Figure S15** Quantitative immunofluorescence analysis and ratio changes of JC-1 aggregates (red) and JC-1 monomers. *p < 0.05, **p < 0.01, ***p < 0.01, ns, nonsignificant.


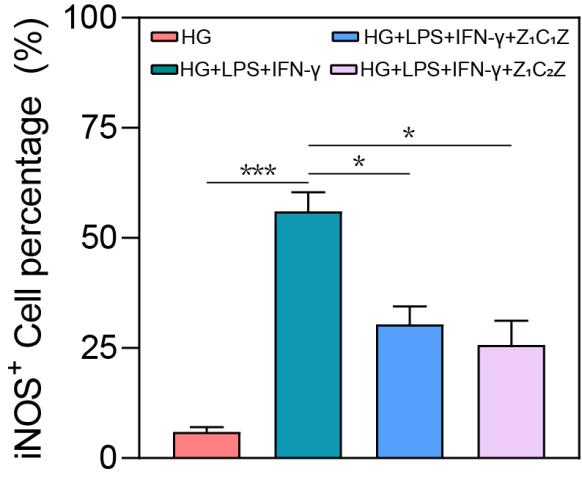


**Figure S16** Statistical analysis of immunofluorescence of iNOS in M1 macrophages. *p < 0.05, ***p < 0.01.

**
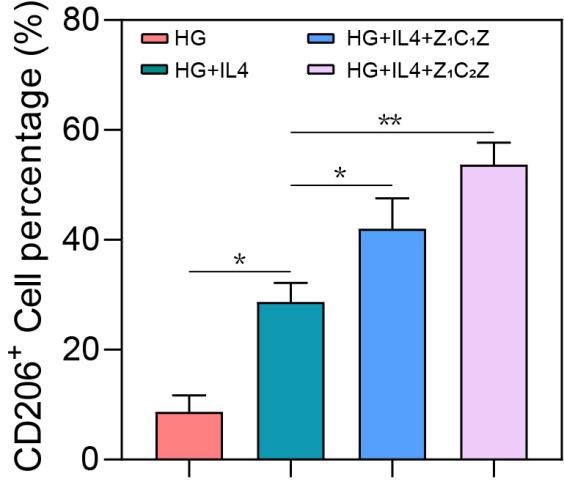
**

**Figure S17** Statistical analysis of immunofluorescence of CD206 in M2 macrophages. *p < 0.05, **p < 0.01.


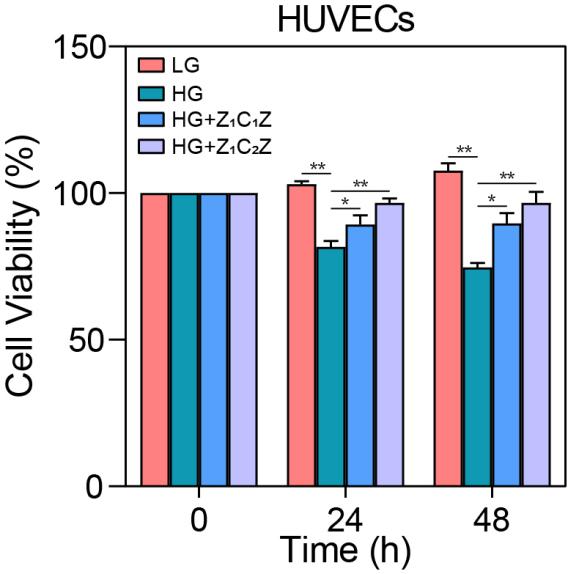


**Figure S18** CCK8 analysis of HUVECs cell viability in HG environment with different treatments. *p < 0.05, **p < 0.01.


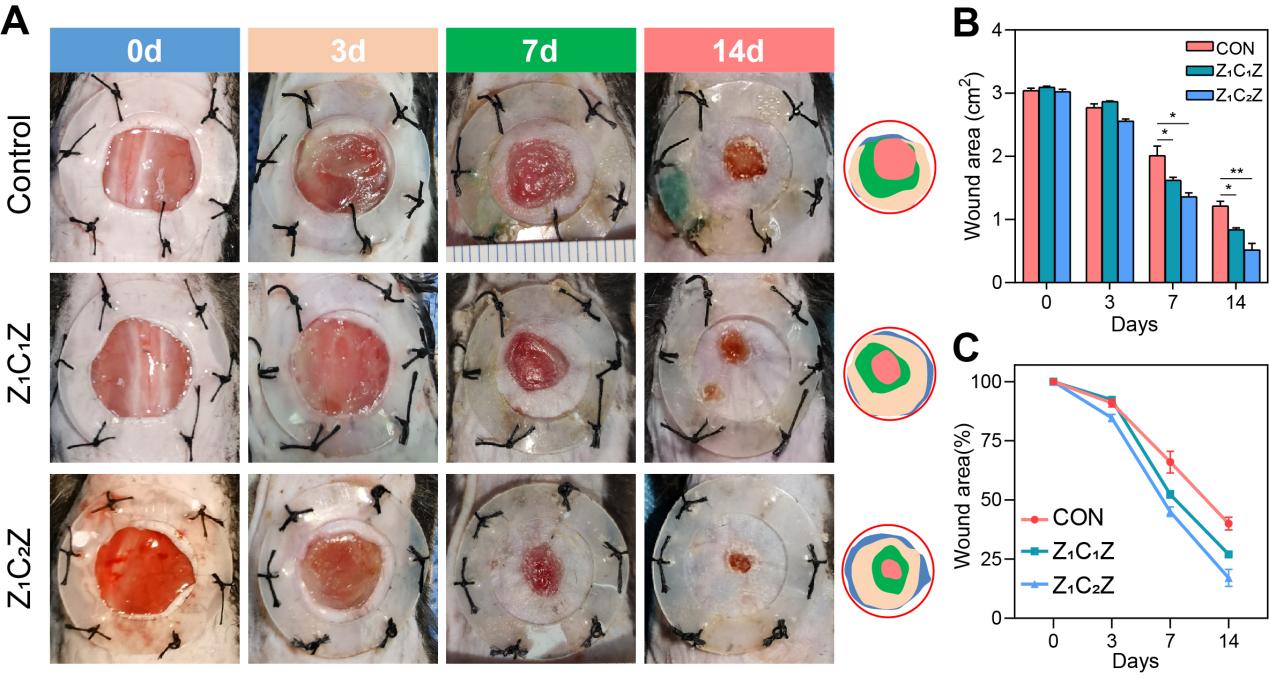


**Figure S19 Z1C2Z promoted wound healing in normal C57 mice** (A) Follow-up images of wound healing in normal C57 mice treated with Z_1_C_1_Z and Z_1_C_2_Z for 2 weeks in vivo. Quantitative analysis of the area (B) and percentage (C) of residual wound after 2 weeks of different treatments. Data are mean standard error of the mean, p values are based Student’s t test. *p < 0.05, **p < 0.01.


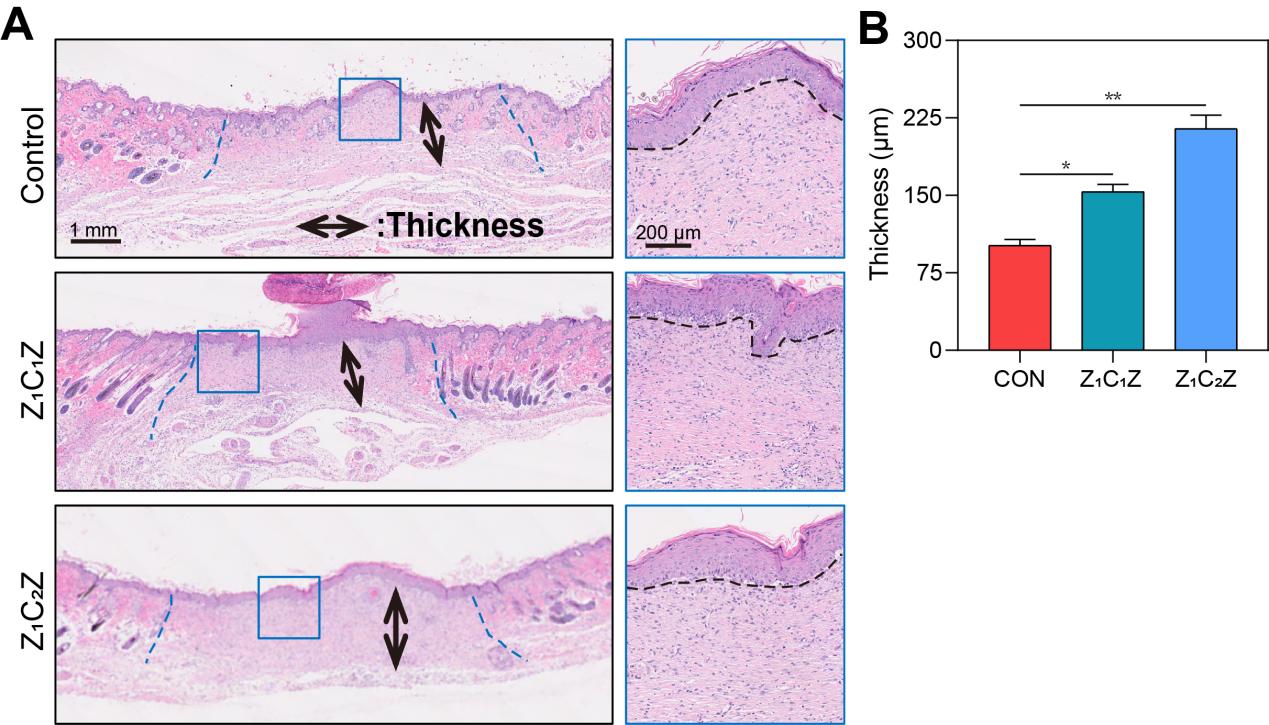


**Figure S20** Representative HE staining images of wound healing and dermal thickness after different treatments for 2 weeks. *p < 0.05, **p < 0.01.


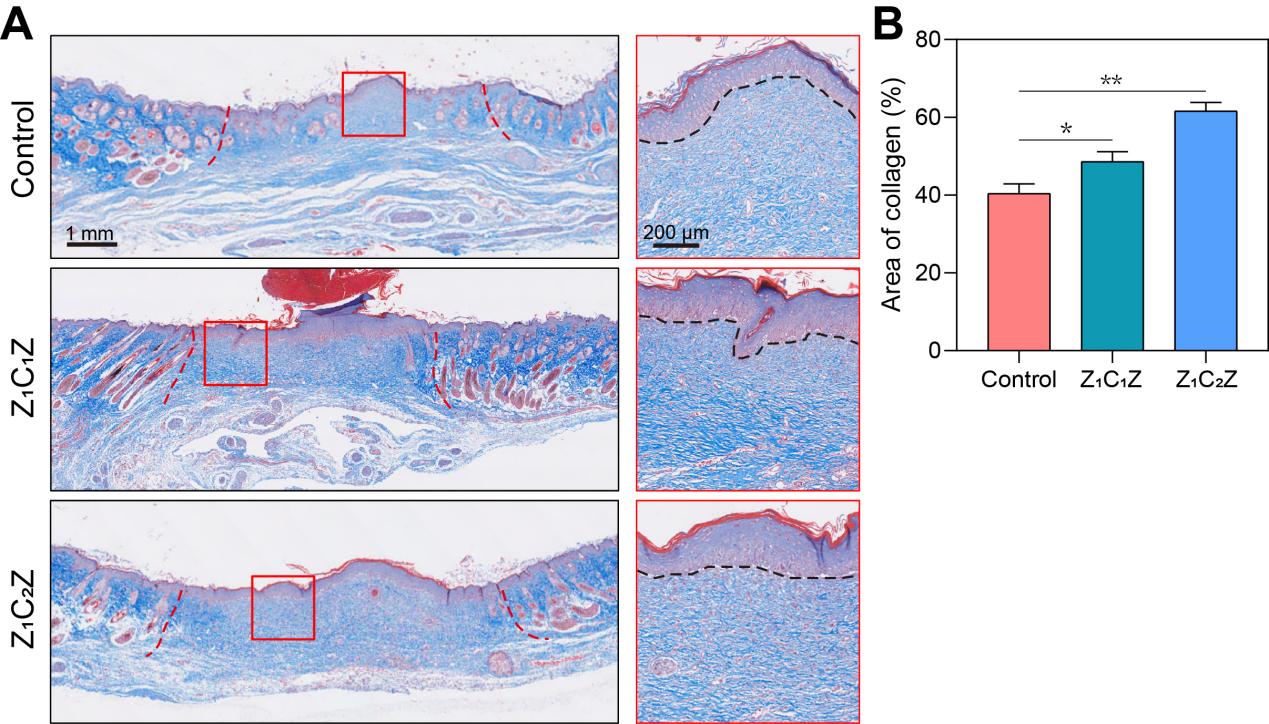


**Figure S21** Representative masson's trichrome staining images of wound healing and collagen content measurement after different treatments for 2 weeks. *p < 0.05, **p < 0.01.


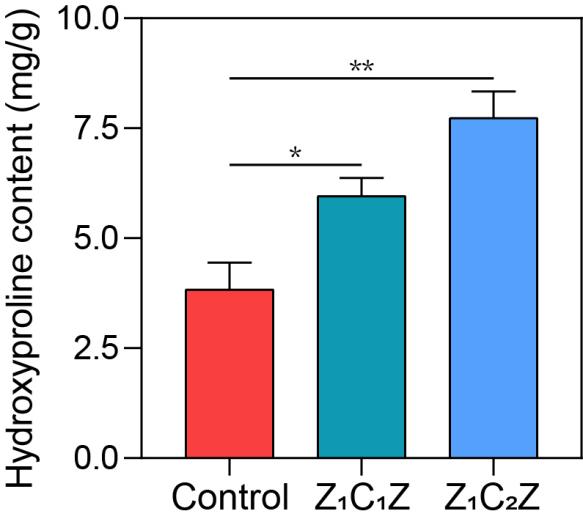


**Figure S22** Quantitative analysis of hydroxyproline determination in different C57 mouse groups. *p < 0.05, **p < 0.01.


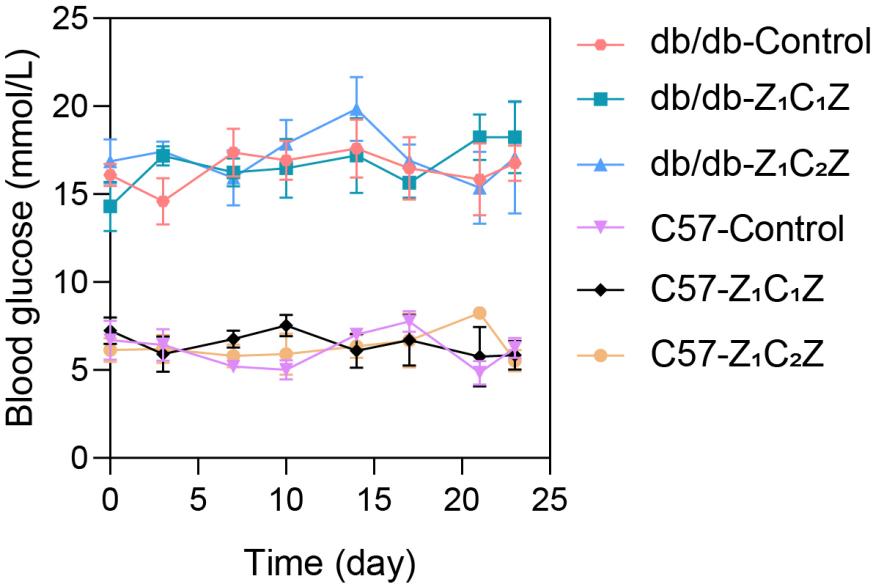


**Figure S23** Fasting blood glucose level changes in db/db mice after different treatment.


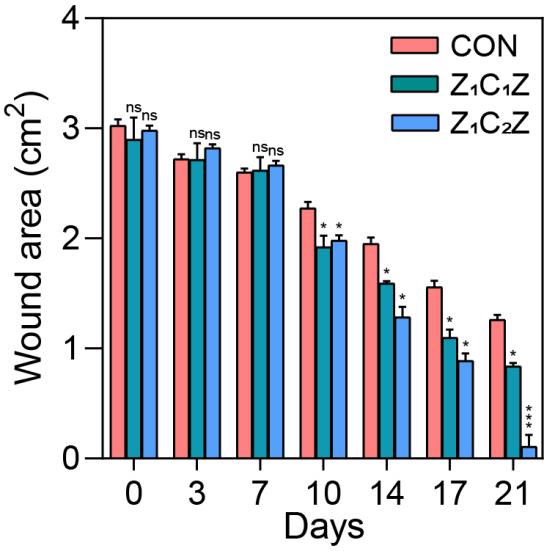


**Figure S24**Quantitative analysis of the area of residual wound during 3 weeks of different treatments. Data are mean standard error of the mean, p values are based Student’s t test. *p < 0.05, **p < 0.01, ***p < 0.01, ns, nonsignificant.


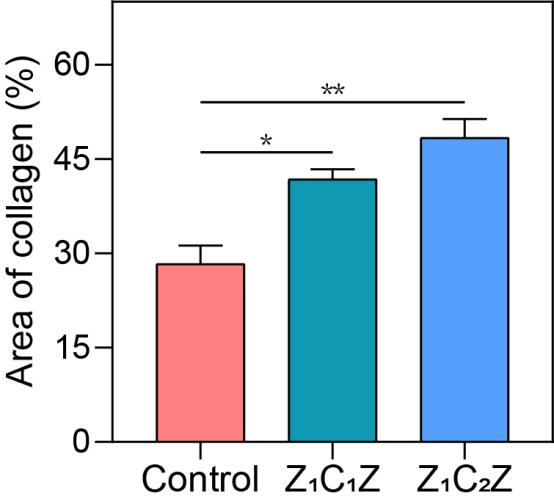


**Figure S25** Quantitative analysis of collagen content throuth masson staining in different diabetic mice groups. *p < 0.05, **p < 0.01.


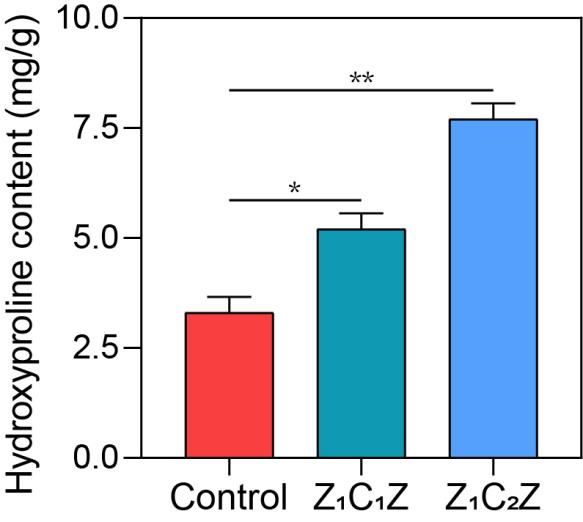


**Figure S26** Quantitative analysis of hydroxyproline determination in the dermis of different db/db mice groups after complete healing. *p < 0.05, **p < 0.01
